# Emergent mechanical control of vascular morphogenesis

**DOI:** 10.1126/sciadv.adg9781

**Published:** 2023-08-11

**Authors:** Jordan Whisler, Somayeh Shahreza, Karin Schlegelmilch, Nil Ege, Yousef Javanmardi, Andrea Malandrino, Ayushi Agrawal, Alessandro Fantin, Bianca Serwinski, Hesham Azizgolshani, Clara Park, Victoria Shone, Olukunle O. Demuren, Amanda Del Rosario, Vincent L. Butty, Natalie Holroyd, Marie-Charlotte Domart, Steven Hooper, Nicolas Szita, Laurie A. Boyer, Simon Walker-Samuel, Boris Djordjevic, Graham K. Sheridan, Lucy Collinson, Fernando Calvo, Christiana Ruhrberg, Erik Sahai, Roger Kamm, Emad Moeendarbary

**Affiliations:** ^1^ Department of Mechanical Engineering, Massachusetts Institute of Technology, Cambridge, MA, USA.; ^2^ Department of Mechanical Engineering, University College London, London, UK.; ^3^ Tumour Cell Biology Laboratory, Francis Crick Institute, London, UK.; ^4^ Mnemo Therapeutics, 101 Boulevard Murat, 75016 Paris, France.; ^5^ Department of Biological Engineering, Massachusetts Institute of Technology, Cambridge, MA, USA.; ^6^ Biomaterials, Biomechanics and Tissue Engineering Group, Department of Materials Science and Engineering and Research Center for Biomedical Engineering, Universitat Politècnica de Catalunya (UPC), Av. Eduard Maristany, 10-14 08019 Barcelona, Spain.; ^7^ UCL Institute of Ophthalmology, University College London, London, UK.; ^8^ Department of Biosciences, University of Milan, Via G. Celoria 26, 20133 Milan, Italy.; ^9^ 199 Biotechnologies Ltd., Gloucester Road, London W2 6LD, UK.; ^10^ Northeastern University London, London, E1W 1LP, UK.; ^11^ Experimental Histopathology Laboratory, Francis Crick Institute, London, UK.; ^12^ Department of Biology, Massachusetts Institute of Technology, Cambridge, MA, USA.; ^13^ David H. Koch Institute for Integrative Cancer Research, Massachusetts Institute of Technology, Cambridge, MA, USA.; ^14^ UCL Centre for Advanced Biomedical Imaging, Paul O'Gorman Building, 72 Huntley Street, London, UK.; ^15^ Electron Microscopy Laboratory, Francis Crick Institute, London, UK.; ^16^ Department of Biochemical Engineering, University College London, London, UK.; ^17^ School of Life Sciences, Queen’s Medical Centre, University of Nottingham, Nottingham, UK.; ^18^ Instituto de Biomedicina y Biotecnología de Cantabria (Consejo Superior de Investigaciones Científicas, Universidad de Cantabria), Santander, Spain.

## Abstract

Vascularization is driven by morphogen signals and mechanical cues that coordinately regulate cellular force generation, migration, and shape change to sculpt the developing vascular network. However, it remains unclear whether developing vasculature actively regulates its own mechanical properties to achieve effective vascularization. We engineered tissue constructs containing endothelial cells and fibroblasts to investigate the mechanics of vascularization. Tissue stiffness increases during vascular morphogenesis resulting from emergent interactions between endothelial cells, fibroblasts, and ECM and correlates with enhanced vascular function. Contractile cellular forces are key to emergent tissue stiffening and synergize with ECM mechanical properties to modulate the mechanics of vascularization. Emergent tissue stiffening and vascular function rely on mechanotransduction signaling within fibroblasts, mediated by YAP1. Mouse embryos lacking YAP1 in fibroblasts exhibit both reduced tissue stiffness and develop lethal vascular defects. Translating our findings through biology-inspired vascular tissue engineering approaches will have substantial implications in regenerative medicine.

## Teaser

Stromal cells create a biomechanically suitable environment for endothelial cells to form microvascular networks.

## INTRODUCTION

The formation of functional vasculature involves complex interactions between endothelial cells (ECs), stromal cells, and extracellular matrix (ECM) (*1*, *2*). Prior work has focused on the spatiotemporal presentation of proangiogenic growth factors and their downstream effects on biochemical signaling pathways (*3*). In particular, vascular endothelial growth factor (VEGF) drives changes in cell shape and, via interaction with Notch signaling, helps specify tip and stalk cells during the growth and morphogenesis of vascular networks. In addition, vascular cells are also mechanosensitive (*4*, *5*), and external mechanical cues regulate vascular processes (*6*–*9*). For example, fluid shear stress (*10*, *11*), substrate stiffness (*12*–*14*), and forces (*15*–*18*) regulate angiogenesis, vasculogenesis, and vascular pathologies. The cell adhesion molecule platelet EC adhesion molecule–1 (PECAM1)/CD31 interacts with VEGFR2 to sense alterations in fluid flow and modulates the function of CDH5/VE-cadherin and, hence, endothelial cell-cell junctions (*19*–*21*). In addition, ECs are highly sensitive to cell matrix adhesions (*22*). Mechanoresponsive transcriptional regulators including Yes-Associated Protein 1 (YAP1) and WW Domain Containing Transcription Regulator 1 (TAZ/WWTR1) (*23*, *24*) respond to both cell-cell and cell-matrix cues and are required within ECs for effective vascular function (*25*, *26*). Both the development and maintenance of vascular networks are highly dependent on supporting mural cells. Pericytes, specialized mesenchymal cells, play a key role in providing both soluble and mechanical cues that support the integrity of EC networks lining blood vessels (*27*). Recent work has shown a role for YAP1 and TAZ/WWTR1 in pericytes in supporting both epithelial and EC function in the lungs (*28*). However, their role in mesenchymal cells during the growth of vascular networks, both in vitro and in vivo, is not understood. More generally, little is known about emergent mechanisms that generate mechanical cues intrinsic to the developing vasculature and control vascular morphogenesis. How an initially mechanically homogeneous embryo gives rise to tissues with highly divergent mechanical properties is not well understood. Here, we combine the use of microfluidic three-dimensional (3D) cultures composed of ECs and fibroblasts with the analysis of vascularization in mouse embryos to reveal the emergent mechanical control of vascular morphogenesis and show that this is critically dependent on YAP1 in mesenchymal cells.

## RESULTS

### Vascular network formation is accompanied by tissue stiffening

**Fig. 1. F1:**
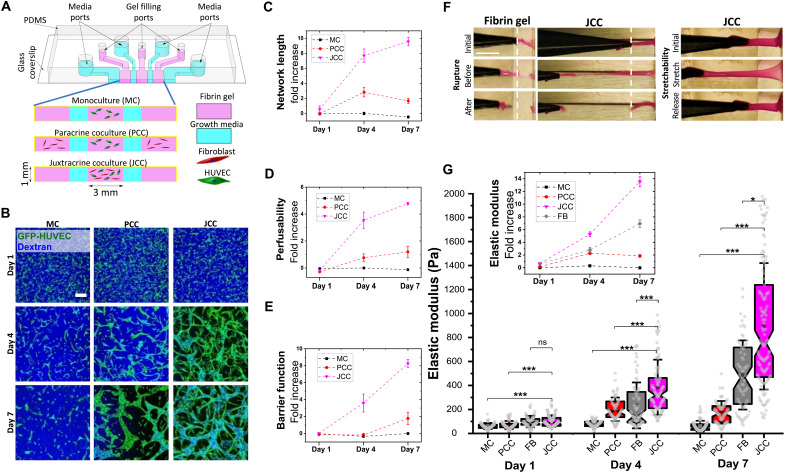
Emergence of enhanced stiffening through juxtacrine interactions. (**A**) Seven-channel polydimethylsiloxane (PDMS) device to study microvasculature formation with three parallel hydrogel channels separated by growth medium perfusion channels. MC condition: ECs encapsulated in central vascularization hydrogel channel and cell-free hydrogels in side channels; PCC condition: ECs encapsulated in vascularization channel and fibroblasts encapsulated in side channels; JCC condition: ECs and fibroblasts encapsulated together in vascularization channel and cell-free hydrogels in side channels. (**B**) Fluorescent dextran perfusion assay. For fully formed vasculature, dextran flows through the vessel lumens and remains within vessel walls (i.e., JCC days 4 and 7). For unconnected vascular structures, dextran flows freely into the fibrin gel (i.e., MC days 1, 4, and 7). Scale bar, 200 μm. (**C** to **E**) Fold increases (means ± SEM) in length of the longest connected network, perfusability, and barrier function compared to MC day 1. (**F**) Manual stretching of fibrin gel or JCC vascularized tissue constructs (day 7) removed from device and anchored at one end while clamped and pulled at the other end. JCC tissue withstood extensive stretch until rupture (300% versus 100% for fibrin gel) and could be reversibly stretched by up to ~100%. Scale bar, 5 mm. (**G**) Apparent elastic modulus of the vascularized tissue, measured by AFM indentation. FB condition: fibroblasts encapsulated in central vascularization channel and cell-free hydrogels in side channels. Inset: Fold increases (means ± SEM) in elastic modulus compared to stiffness of MC day 1. At least 50 AFM indentation measurements on *N* = 3 independent devices were performed per condition. Kruskal-Wallis test followed by Dunn’s multiple comparison test: *P < 0.01 and ***P < 0.0001 (table S1). ns, not significant.

To study the mechanics of vascular morphogenesis systematically, we adapted an in vitro microfluidic platform (*29*, *30*) to enable the formation of tissue-scale constructs for mechanical characterization and observation of vascular self-assembly in various coculture configurations of ECs [human umbilical vein ECs (HUVECs)] with stromal fibroblasts (normal human lung fibroblasts; Fig. 1A and fig. S1, A and B; detailed description in Materials and Methods). Specifically, ECs were embedded within a fibrin gel in a 3D tissue vascularization channel under three conditions: either alone in a monoculture (MC), spatially separated from fibroblasts by a growth medium perfusion channel [“paracrine coculture” (PCC)], or in direct contact with fibroblasts [“juxtacrine coculture” (JCC)] (Fig. 1A). In the MC condition, ECs initially elongated and self-assembled into multicellular structures, but intercellular connections were subsequently lost, and the structures regressed after 96 hours (Fig. 1B). In contrast, extensive vascular networks were formed in the JCC condition (Fig. 1, B and C). The PCC condition resulted in a vascular network that was more developed than MC but less developed than JCC. We quantified functional properties of the self-assembled vasculature by introducing a fluorescent dextran tracer into the perfusion channel (fig. S1, C to F). The JCC condition resulted in vascular networks that were highly perfusable (note the strong dextran signal within the vessels) and had enhanced barrier function (note the weak dextran signal within the interstitial space) compared to either MC or PCC (Fig. 1, B, D, and E).

The tissue constructs in our model could be removed from the vascularization channel to perform mechanical testing by manual stretching or atomic force microscopy (AFM). In the absence of cells, the extracted fibrin tissue constructs easily disintegrated when minimal external forces were manually applied, demonstrating a mechanical response akin to soft hydrogel (Fig. 1F). In contrast, when ECs and fibroblasts were cultured within the fibrin for 7 days, the tissue constructs maintained their structural integrity when stretched (Fig. 1F). Specifically, the fully vascularized tissue constructs (JCC condition) exhibited reversible deformation for applied strains of up to 100% (Fig. 1F, right). To quantify the emergent mechanical properties of the vascularized tissue constructs more precisely, we performed AFM indentation tests. In JCC, stiffness increased over the course of 1 week, from 100 Pa at day 1 to over 900 Pa at day 7 (Fig. 1G). For the PCC condition, the stiffness increase was less pronounced, reaching an average of 270 Pa at day 7, while for MC, no stiffening was observed (Fig. 1G). The stiffness of constructs containing monoculture fibroblasts did increase but was significantly lower compared to JCC (*P* = 0.00121; Fig. 1G, condition FB). These results suggest that vascular network formation driven by juxtacrine interactions between ECs and fibroblasts is accompanied by notable changes in tissue mechanics.

### Juxtacrine interactions shape the unique vascular tissue mechanics

**Fig. 2.**
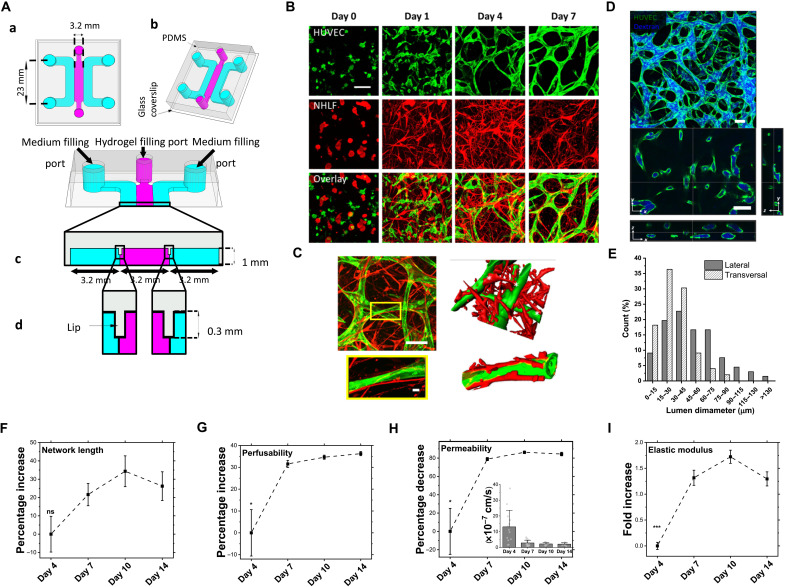
Temporal correlation of stiffness with morphological and functional characteristics. (**A**) Three-channel PDMS device comprising a central vascularization channel (pink) and two growth medium perfusion channels (blue). (a and b) 2D and 3D views. (c) Channel dimensions. (d) PDMS lips extending from the top surface prevent leakage of prepolymerized hydrogel from central vascularization channel into medium perfusion channels via surface tension. (**B**) Confocal imaging of vascular morphogenesis progression for green fluorescent protein (GFP)–HUVECs and red fluorescent protein–fibroblasts in JCC condition. Scale bar, 100 μm. (**C**) Visualization of EC-fibroblast interactions. Scale bar, 100μm (top left). Bottom left: Magnified image of the yellow box showing fibroblasts extending along the outside of the EC vessel wall. Scale bar, 10 μm. Right images are 3D reconstructions of left images. Top right: A fibroblast extending toward multiple vessels. * denotes region of interaction. (**D**) *Z*-projected confocal image of GFP-HUVECs in JCC system perfused with blue fluorescent dextran on day 7. Bottom images are orthogonal views showing lateral (parallel to glass substrate) and transversal (perpendicular to glass substrate) cross sections with tracer contained within vascular lumens. Scale bars, 100 μm. (**E**) Distribution of lumen diameters on day 7, measured using images in (D). (**F** to **H**) Temporal percentage changes (means ± SEM) in network length, perfusability, and permeability, normalized to day 4 values. Inset in (H) shows the permeability values (means ± SEM). (**I**) Temporal fold increase (means ± SEM) in stiffness of the vascular tissue normalized to day 4. For (F to I), measurements were performed on *N* = 3 independent devices for each time point. Kruskal-Wallis test followed by Dunn’s multiple comparison test for day 4: *P < 0.01 and ***P < 0.0001 (table S2).

To unravel the origins of enhanced intrinsic stiffening in more detail and its temporal progression, we developed a simplified microfluidic device in which the vascular tissue construct could be maintained for up to 14 days (Fig. 2A). Imaging revealed profound morphological changes in both ECs and fibroblasts over the first week (Fig. 2B). ECs were initially rounded and monodispersed but then spread out and extended protrusions that developed into intercellular connections before forming a perfusable network by day 4 (Fig. 2B). From days 4 to 7, the overall network structure remained stable, while individual vessel branches continued to mature, forming smooth vessel boundaries with well-defined lumens (Fig. 2B; fig. S2, A and B; and Supplementary Notes). Fibroblasts also underwent remarkable and rapid shape changes; already by day 1, they extended protrusions and became elongated, with some individual cells spanning several hundred micrometers (Fig. 2B). By day 7, fibroblasts appeared to be preferentially concentrated around the vasculature (Fig. 2B). Notably, we found evidence of direct, physical interaction, characterized by the elongation of fibroblasts along the external vessel walls (Fig. 2C and fig. S3), as observed in vivo (*31*). Comparison of MC, PCC, and JCC conditions, including the evaluation of conditioned media and cell density confirmed the critical importance of juxtacrine interactions (fig. S4).

**Fig. 3.**
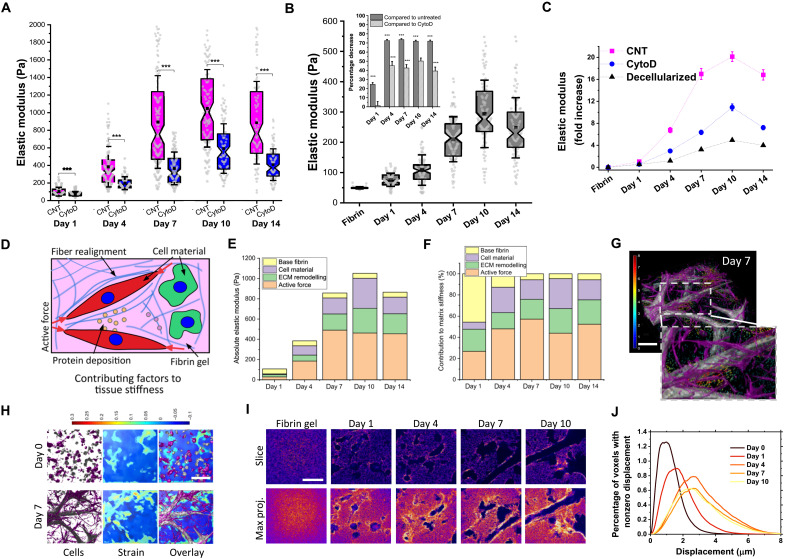
Origins of vascular tissue stiffening and evolution of strains. (**A**) Vascular tissue stiffening over time for JCC control (CNT) and the softening effect of cytochalasin D (CytoD) treatment. Minimum of 50 AFM indentation measurements on *N* = 3 independent devices before and after treatment for each time point. Two-sided Mann Whitney *U* test, ***P < 0.0001. (**B**) Stiffness of the decellularized tissue. The inset shows percentage decrease (means ± SEM) in the stiffness of decellularized tissue compared to the stiffness of either untreated or cytochalasin D–treated vascular tissue at each time point. Two-sided Mann Whitney *U* test, ***P < 0.0001. (**C**) Fold increase (means ± SEM) in stiffness of vascularized tissue constructs for control (CNT), cytochalasin D, or decellularized treatments compared to cell-free fibrin gel. (**D**) Contribution of different factors to the tissue stiffness. (**E**) Absolute and (**F**) relative contribution of each factor. (**G**) 3D reconstructed vascularized region (size of 420 μm by 420 μm by 80 μm) showing fibroblasts (magenta) and HUVECs (gray) with superimposed, color-coded (0 to 8 μm) arrows that depict the magnitude and direction of 3D matrix displacements obtained by tracking fluorescently labeled fibrin gel. (**H**) Cross-sectional view of the 3D stack from (G) showing cellular structures (left; fibroblasts in magenta and HUVECs in gray), matrix strain field (center), and the overlay (right). Scale bar, 100 μm. (**I**) Confocal images of fibrin gel fiber structures acquired after decellularization. Thick bundles of fibers were observed at the edges of voids previously occupied by cellular structures. Scale bar, 100 μm. (**J**) Histograms of nonzero displacement magnitudes (induced by cytochalasin D treatment) and their relative frequencies for different time points (curves are averages of three regions).

Using dextran perfusion and previously described methods (*32*, *33*), we measured individual vessel diameters spanning a range of 5 to 150 μm (Fig. 2, D and E) and calculated average vessel diameter in both lateral (44 ± 5 μm) and transversal (30 ± 3 μm) directions, leading to an average hydraulic diameter of 34 ±20 μm for day 7 JCC systems. These measurements correspond to a physiological microvasculature composed of capillaries (5 to 10 μm in diameter), venules, and arterioles (10 to 100 μm in diameter) (*34*, *35*). The similarity of the size distributions in lateral and vertical directions indicates a uniform distribution of vessels throughout the 3D tissue construct. We also measured individual vessel lengths spanning a range of 50 to 900 μm with an average length of 90 ± 5 μm (fig. S2C). Tracking of vessel metrics from day 4 to day 14 indicated that the network length did not undergo large-scale changes from day 4 through at least day 14 (Fig. 2F). Measurements of perfusability and permeability revealed that the JCC network increased in functionality between day 4 and day 7 and then transitioned to a mature and stable phenotype (Fig. 2, G and H). Similarly, the stiffness of the network increased markedly from day 4 to day 7 (Fig. 2I). Notably, establishment of the functionally stable vascular network beyond day 7 was correlated to achieving mechanically stable vascular tissue (Fig. 2, G to I). These observations that the most marked changes in vascular network features including length, perfusion, permeability, and stiffness occur within the first 7 days are consistent with previous works (*33*, *36*), demonstrating the criticality of the initial few days of vascular morphogenesis. Slight decreases in network length and stiffness were observed at day 14, but these reductions were not statistically significant when compared collectively to previous time points (days 7 and 10). Last, measurements of vascular density (46 ± 5 vessels/mm^2^; fig. S2D), vascular volume fraction (14 ± 3%; fig. S2E), vascular permeability (~10^−7^ cm/s; Fig. 2H inset), and vascular stiffness (~1 kPa; Fig. 3A) were all concordant with previously reported physiological values (*33*, *37*–*40*), providing a high level of confidence in the physiological relevance of our in vitro model. Together, these data demonstrate a strong correlation between metrics of vascular function and tissue stiffness during the development and maintenance of a functional vascular network.

We additionally investigated whether tissue stiffening was associated with the vascularization of the mouse retina, a widely studied model of angiogenesis. Measurements of the spatial distribution of stiffness along the retina radius (fig. S5A; R1 to R3) revealed that areas devoid of vessels at postnatal day 3 (P3) were softer compared to vascularized regions (fig. S5B; compare region R1 with vessels to R3 without vessels). Furthermore, as vascular development progressed, the stiffness of the vascularized tissue increased significantly, with the maximum stiffness achieved in R2 at P7 (fig. S5B). These findings demonstrate that emergent stiffening is a mechanical signature of vascular tissue development and maturation in vivo and further support the relevance of our in vitro JCC system for studying the mechanics of tissue vascularization.

### Active cellular force generation is the major contributor to tissue stiffening

Having established the emergence of enhanced tissue stiffening, we investigated the possible cause of the stiffening. Specifically, we constructed a model to evaluate the contribution of active forces applied by cells and changes in ECM mechanics (*41*–*44*). This highlighted that cell-generated forces could play a major role in the emergent stiffening of the vascular networks (fig. S5, C to F, and Supplementary Notes). To measure their contribution, we inhibited the ability of the cells to generate active forces via their actin cytoskeleton. Following the targeting of the actin network with cytochalasin D, the average stiffness of vascularized tissue constructs was significantly reduced (Fig. 3A), but it did not revert to the tissue stiffness levels observed at day 1. Furthermore, the stiffness of decellularized tissue constructs increased progressively (Fig. 3, B and C), but for any given time point (after day 1), the decellularized tissue constructs were approximately 70% softer than the corresponding live cellularized tissue constructs (Fig. 3B, inset). In our system, tissue stiffness is influenced by four main factors: initial fibrin hydrogel, cellular material, ECM remodeling, and active stiffening due to cell-generated forces (Fig. 3D). As vascular morphogenesis progresses, active stiffening emerges as the dominant single contributor to overall stiffness with smaller but still considerable contributions from cellular material and ECM remodeling (Fig. 3, E and F). Furthermore, similar results were obtained when analyzing mouse retina. Treatment of retinal tissues with cytochalasin D perturbed the force generation ability of all cell types within retinal tissue (i.e., ECs, astrocytes, and pericytes) and resulted in a significant decrease in tissue stiffness at vascularized regions at P7 (fig. S5B).

Because our data establish active cellular forces to be central to the tissue stiffening associated with the formation of vascular networks, we next determined the distribution and magnitude of active forces generated by cells in the JCC system by conducting 3D traction force microscopy (Fig. 3G). At the very early stages of culture (days 0 and 1), the strain distribution resembled that of a typical cell-embedded fibrous biopolymer (*45*, *46*), with strains concentrated along paths of interaction between individual cells (Fig. 3H). However, in the mid- to late stages of culture (from day 4 onward), high levels of traction strains emerged around the lumenized vascular structures (Fig. 3H). High levels of traction strains proximal to vessels were also associated with increased fibrin density around the vessels (Fig. 3I). Histograms of nonzero displacement magnitude and their relative frequency show that the magnitude of deformations progressively increased throughout the course of vascular morphogenesis exhibiting over 6-μm displacements after day 7 (Fig. 3J). This trend correlates with the observed progressive stiffening. The pattern of remodeled matrix fibers was parallel to the structural organization of the vasculature (Fig. 3I), suggesting that vascularized tissue mechanics may resemble those of a fiber-reinforced composite with both active (cellular contractile forces) and passive (ECM fiber bundles) elements contributing to the effective stiffness (*47*). In summary, these data demonstrate that ~50% of stiffening during vascular network formation is driven by active cellular forces (Fig. 3F), which synergize with the structural changes in the ECM to achieve up to ~20-fold overall stiffening of the vascularized tissue compared to nonvascularized fibrin gel (Fig. 3C).

### Intrinsic stiffening is regulated by fibroblasts through YAP1-dependent mechanotransduction

To determine how reciprocal signaling between ECs and fibroblasts might lead to changes in tissue mechanics, we performed RNA sequencing on ECs and fibroblasts in JCC and MC conditions. Principal components analysis indicated progressive changes in the transcriptome of both ECs and fibroblasts as the culture matured (fig. S6, A and B). Gene set enrichment analysis (GSEA) revealed that these changes affected EC proliferation and differentiation genes, including many involved in Notch and bone morphogenetic protein signaling (fig. S6C). The poor viability of HUVEC after 14 days in MC precluded analysis of the genes specifically induced by juxtacrine coculture with fibroblasts at that time point. JCC triggered a notable up-regulation of genes in fibroblasts associated with contractile myofibroblasts (myCAF) and mechanotransduction (YAP1 regulated up; fig. S6D). Genes involved in inflammatory signaling were down-regulated over the same period.

**Fig. 4.**
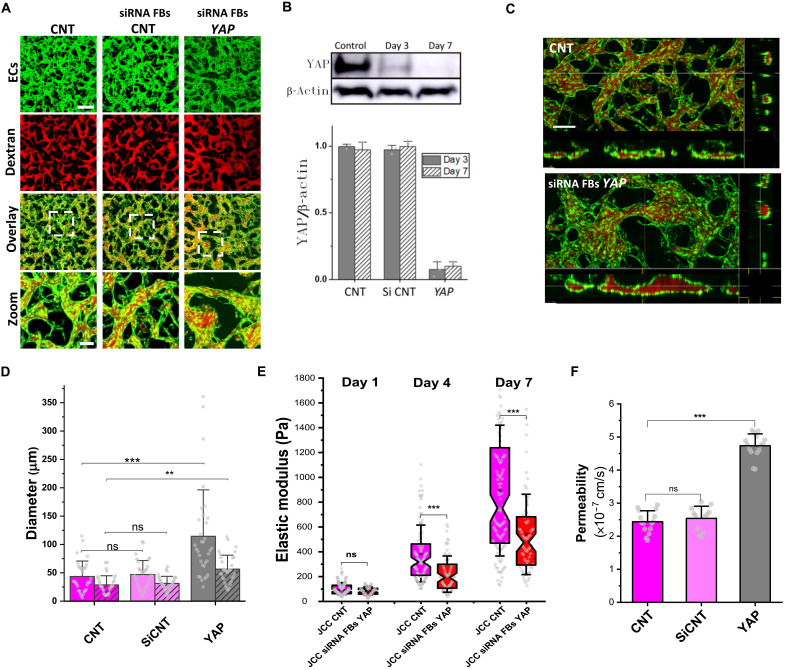
Fibroblasts control vascular morphogenesis through YAP1. (**A**) Fibroblasts were transfected with siRNAs targeting YAP1 before culture in the JCC system. Confocal images showing vasculature (GFP-HUVECs) perfused with dextran (red) in JCC control (CNT) and JCC fibroblast YAP knockdown (siRNA FBs YAP) conditions at day 7. The network perfusability and morphology were unaffected by scrambled siRNA (siRNA FBs CNT) but perturbed to varying degrees by siRNA knockdown. Scale bars, 200 μm and 50 μm (zoomed images). (**B**) Western blots of YAP protein in fibroblasts before siRNA transfection (Control) and at 3 and 7 days after transfection. The bar graph (bottom) shows quantification of transfection efficiency. Fibroblast YAP expression levels were normalized to β-actin values for each condition and time point before making comparisons. (**C**) Confocal images showing *z* projection and orthogonal slices of vasculature (GFP-HUVEC) perfused with dextran (red). Scale bar, 100 μm. (**D**) Quantification of lateral (unshaded bars) and transversal (shaded bars) diameters (means ± SD) comparing siRNA FBs YAP, CNT and siRNA FBs CNT at day 7 using images from (C). Measurements were performed on *N* = 4 devices from two separate experimental repeats. Linear analysis of variance (ANOVA) followed by Tukey-Kramer test, **P < 0.001 and ***P < 0.0001. (**E**) Stiffness of the vascular tissue constructs over time, comparing siRNA FBs YAP to CNT. Minimum of 50 AFM indentation measurements for *N* = 3 devices from two separate experimental repeats for each condition and time point. Kruskal-Wallis test followed by Dunn’s multiple comparison test, ***P < 0.0001. (**F**) Permeability (mean ± SD) at day 7 comparing siRNA FBs YAP, CNT and siRNA FBs CNT. Measurements on *N* = 4 devices from two separate experimental repeats. Linear ANOVA followed by Tukey-Kramer test, ***P < 0.0001.

Considering the above gene expression analyses together with existing literature on YAP1 and its links to active force generation by the actin network (*48*–*51*), we tested the functional relevance of this pathway for vascular morphogenesis in our vascularized tissue constructs. First, we blocked the function of YAP1 for both cell types using the chemical inhibitor verteporfin, which led to large-scale defects in vascular organization without compromising cell viability (fig. S7, A to C). This result was consistent with previous studies that demonstrated the critical role of YAP activation, mainly within ECs, to promote angiogenesis (*23*–*26*). Having shown thus far, in our study, the importance of juxtacrine interactions with fibroblasts, we next investigated whether YAP signaling in fibroblasts might independently play a role in vascular morphogenesis by depleting YAP1 using small interfering RNA (siRNA) solely in fibroblasts before inclusion in vascular tissue constructs. The efficiency of the knockdown was confirmed by Western blotting (Fig. 4B) and through quantification of immunofluorescence staining for YAP1 in devices at day 7 (fig. S8, A and B). YAP1 depletion in fibroblasts led to substantial changes in vascular network morphology (Fig. 4A; see zoomed in images at the bottom),as evidenced by increased vessel diameter in both lateral and transversal directions (Fig. 4, C and D). Stiffness measurements during the first week of vascular morphogenesis revealed that depleting YAP1 in fibroblasts led to a notable reduction in the elastic modulus of the tissue. At day 7, the stiffness of JCC tissue constructs containing fibroblasts depleted for YAP1 was decreased by over 40% compared to control (Fig. 4E). This correlated with decreased barrier function as measured by an increase in permeability when YAP1 was depleted in fibroblasts (from 2.4 × 10^−7^ to 4.7 × 10^−7^ cm/s; Fig. 4F). Immunofluorescence staining also revealed lower levels of VE-cadherin expression in ECs for the JCC FB^-YAP^ system (fig. S8, A and C). These data establish YAP1 in fibroblasts as a key regulator of the active stiffening required for effective vascular network formation.

### Loss of YAP1 in fibroblasts leads to lethal vascular defects

**Fig. 5.**
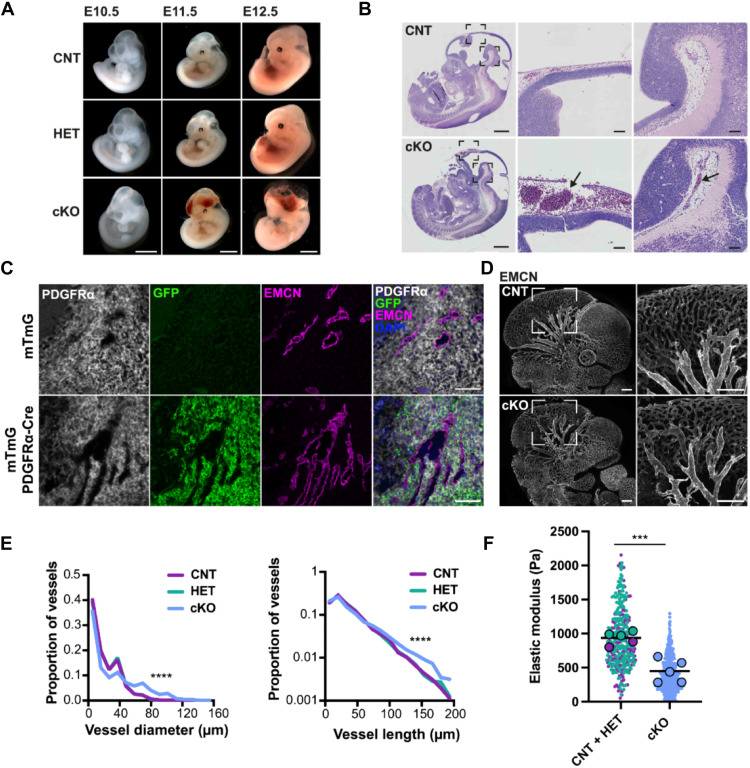
Loss of YAP1 in fibroblasts is embryonic lethal because of vascular defects. (**A**) Isolation of control (CNT), YAP^fl/+^ PDGFRα-Cre positive (HET), and YAP^fl/+^ PDGFRα-Cre positive (cKO) embryos at different embryonic stages shows a hemorrhage in cKO embryos at E11.5 and E12.5. Scale bars, 2 mm. (**B**) Hematoxylin and eosin images of E12.5 embryos show that defects in cKO embryos are restricted to the head, with high magnification panels showing vessel distension around the brain in cKO embryos. Black arrows point to erythrocytes. Scale bars, 1 mm and 100 μm in the low and high magnification panels, respectively. (**C**) Images show PDGFRα, GFP, and endomucin (EMCN) staining of the region of the head below the brain in control and PDGFRα-Cre × mTmG mice at E11.5. DAPI (4′,6-diamidino-2-phenylindole) stains nuclei. Scale bars, 100 μm. (**D**) Confocal microscopy of whole-mount immunofluorescence for endomucin (EMCN) with a focus on the central vessels in the head (white square; magnification to the right). Scale bars, 250 μm. (**E**) Quantification of vessel diameter (in μm) and vessel length (as defined by the distance between vessel branches in μm) on confocal whole-mount data using machine learning approaches. **** indicates different distribution of vessel sizes between cKO (*N* = 3, blue) versus HET (*N* = 2, turquoise) and CNT (*N* = 4, purple) (****P < 0.0001, Kolmogorov-Smirnov test). (**F**) Superplot of AFM data on head region of interest of freshly isolated CNT (*N* = 2, purple), HET (*N* = 3, turquoise), and cKO (*N* = 5, blue) embryos at E11.5. Elastic modulus of cKO was significantly different (***P < 0.001, Nested *t* test).

To explore how mechanotransduction in fibroblasts affects vascular growth in vivo, we crossed YAP1 floxed mice with platelet-derived growth factor receptor alpha (PDGFRA)–Cre expressing mice to generate mice with YAP1-deficient fibroblasts (fig. S9A). PDGFRA is expressed across fibroblastic lineages from embryonic day 6.5 (E6.5). No mice with homozygous deletion of YAP1 in the PDGFRA lineage were born because embryos did not survive beyond E12.5. PDGFRA-Cre YAP1 heterozygous mice were viable and fertile, with no phenotype observed in either males or females (table S3). In PDGFRA-Cre YAP1 floxed homozygous embryos (hereafter referred to as cKO), hemorrhaging was visible in the head region of many embryos but not in the thoracic and abdominal organs (Fig. 5, A and B). Analysis of embryonic tissue with the mTmG reporter confirmed activity of Cre in fibroblasts, but not ECs, in the heads of embryos (Fig. 5C and fig. S9, B and C). These analyses also revealed minimal Cre-driven recombination in the brain. Consistent with mTmG reporter analysis, YAP1 staining was absent in mesenchymal cells in the head of YAP1 cKO embryos but remained visible in adjacent ECs (fig. S9D). Histological analysis indicated that hemorrhaging occurred around the brain with distended vessels congested with nucleated erythrocytes (Fig. 5B). No thrombi were observed, suggesting that the distension of vessels was not due to vessel blockage. At E11.5, the head region around the brain was rich in fibroblasts, but lack of NG2 and desmin staining suggested that mature pericytes were not yet specified in this region of the embryo (fig. S9E). Transmission electron microscopy revealed that fibroblastic cells were forming close contacts with the ECs, reminiscent of those formed by mature pericytes (fig. S9, F and G). Thus, in the absence of mature pericytes, vascular development at this time depends on fibroblastic precursors in the head.

To analyze the altered vascular architecture in more detail, we performed whole-mount endomucin (EMCN) staining on E10.5 embryos, which revealed quantitative differences in the vascular network morphology (Fig. 5, D and E). Concordant with the in vitro assays, there was an increase in larger-diameter vessels in the head region in mice lacking fibroblastic YAP1 compared to controls with an increase in the number of vessels with diameters larger than 60 μm (Fig. 5E and fig. S9I). Although the branching angles between vessels were unchanged, vessel segments were typically longer in mutants compared to controls (Fig. 5E and fig. S9H). Transmission electron microscopy indicated that the interface between ECs and fibroblastic cells was disrupted in YAP1 cKO embryos (fig. S9, F and G). These data demonstrate that YAP1 is required in mesenchymal cells for the correct organization of ECs and blood vessels.

Data described in Fig. 3 suggest that fibroblast-driven tissue stiffening is largely the result of active forces, with a smaller contribution from ECM changes (Fig. 3F). Histological staining did not show changes in Ki67 (a proliferation marker), fibronectin, collagen I, or collagen IV staining in YAP1 cKO embryos compared to control (fig. S10, A to C). Notably, AFM analysis of E11.5 embryos demonstrated that YAP1 deficiency in fibroblasts reduced the elastic modulus of the head region by 50%, indicating a failure in enhanced tissue stiffening in regions with vascular defects (Fig. 5F). These data argue that YAP1 is required for the active component of tissue stiffening, not the passive component of ECM production. To conclude, the “mechanotransducer” YAP1 is required in mesenchymal cells for tissue stiffening that accompanies vascular network formation and development of integrated vasculature.

## DISCUSSION

We engineered 3D large-scale fluidic devices that allowed us to study tissue-scale mechanics of vascular morphogenesis over a 14-day extended period. The larger tissue volume in our device compared to typical microfluidic systems [3 mm (width) by 21 mm (length) by 1 mm (height) versus 0.5 mm (width) by 15 mm (length) by 0.5 mm (height)] provides several key advantages. As well as allowing for real-time, 3D visualization of vascular network formation and functional characterization in a perfusable system, similar to previous microfluidic approaches (*36*, *52*, *53*), our device also enables the removal of intact tissue for mechanical testing and supplies sufficient cell yields to perform robust transcriptional analysis. Thus, we used the platform to monitor the morphological and functional characteristics of vascularized tissue constructs over time, characterize the temporal changes in their mechanical features using AFM and traction force microscopy, and identify a mechanotransduction pathway linking the observations.

The results demonstrate that tissue stiffness progressively increases during vascular self-assembly primarily because of cellular force generation. Furthermore, we showed that physical interaction between ECs and fibroblasts enhances tissue stiffening and promotes the formation of highly connected, perfusable, and stable vascular networks. Our work builds upon previous observations by others, including Juliar *et al.* (*9*) and Friend *et al.* (*54*), linking capillary morphogenesis to matrix stiffening and cell-generated contractile forces. Specifically, using our microfluidic system, we were able to generate a perfusable microvasculature and correlate functional properties such as perfusability and permeability to these mechanical observations. In addition to the emergent tissue stiffening that intrinsically modulates vascular morphogenesis, other extrinsic mechanical factors may regulate vascularization processes. Recently, Zhang *et al.* (*55*) demonstrated that application of interstitial flow improves vasculogenesis through up-regulation of matrix metalloproteinase-2. Consistent with our study, they showed that in the absence of interstitial flow, EC monoculture (even at higher densities compared to ours) cannot lead to the formation of a perfusable network, and the network-like structures regressed. In another study, Song *et al.* (*56*) demonstrated that the presence of fibroblasts during the first 3 days of culture was critical to the formation of functional microvasculature and the effects of coculture persisted even after the fibroblasts were ablated thereafter. Although the specific cell types, seeding densities, and device geometries differ between their study and ours, we similarly found that the contribution of fibroblasts, in our case, through active tissue stiffening, became prominent during the initial 4 days of coculture (see Fig. 3F).

Last, we revealed that the emergent process described herein, which we propose to term “tissue intrinsic stiffening,” is mediated via YAP1 mechanotransduction pathways within fibroblasts and demonstrated that loss of YAP1 in fibroblasts in vivo results in softening of embryonic tissues concomitant with embryonic lethality due to disruption of normal vascular morphogenesis in the head. The manifestation of the vascular phenotype in this anatomical region may be linked to the abrupt transition from large vessel diameters to narrow vessels in the brain leading to high fluid pressures and stresses. The strong nuclear localization of YAP1 in fibroblasts in this region is consistent with a high level of YAP1 function. Previous studies have demonstrated that YAP1 activation is associated with increased contractile force generation by fibroblasts (*50*, *51*), which is consistent with the role that we propose in driving active tissue stiffening. In the future, it will be interesting to determine the signals emanating from ECs that activate YAP1. GSEA analysis suggested a role for Src family kinases in fibroblasts, which is consistent with the known role of Src family kinases in regulating YAP1 transcriptional activity.

Previous studies reported that fibroblastic cells support vascular networks by secreting growth factors (*57*–*59*), synthesizing ECM (*60*, *61*), and stabilizing microvascular networks by physically interacting with the vascular microenvironment (*56*, *62*–*64*). Our study reveals a previously unknown biomechanical mechanism by which fibroblasts promote vascular morphogenesis and function through reciprocal biomechanical interactions with ECs. In vivo, these interactions involve fibroblastic cells before the expression of recognized mural cell markers. These may occur at the extensive sites of contact that we observe in both our models and E11.5 embryos. We propose that increased cytoskeletal tension and tissue stiffening may lead to force-dependent maturation of vascular adherens junctions (fig. S8, A and C), thereby enabling robust vascular integrity. Supporting or even enhancing the mechanical cross-talk between ECs and fibroblasts may be of benefit to treat vascular malformations, promote wound healing, and support the construction of vascularized tissues in situ or for transplantation. One route to achieve this could be positive modulation of YAP1 function, although the positive role of YAP1 in tumorigenesis would mean that this should be locally targeted to the region requiring regeneration and for short durations. To conclude, through combined use of engineered devices and mouse models, we demonstrate a crucial role for YAP1 in the emergent mechanical control of vascular morphogenesis.

## MATERIALS AND METHODS

### Microfluidic platform designs and fabrication

The seven-channel fluidic device (Fig. 1A and fig. S1A) incorporates three cell encapsulation channels. The central vascularization channel is flanked by the other two cell encapsulation channels (one on each side), and all cell channels are separated by a growth-medium-filled perfusion channel. This enables the physical separation of fibroblasts from ECs and guarantees that heterotypic cell-cell communication is limited to the diffusion of secreted factors (PCC condition). In all, the device comprises three hydrogel channels and four perfusion medium channels aligned in parallel (Fig. 1A), each having a rectangular cross section with height equal to 1 mm and width dimensions shown in fig. S1A. A glass coverslip bonded to the bottom of the device enables real-time imaging with standard microscopy techniques. To maximize the steady chemical interaction between fibroblasts and ECs, the width of inner medium channels (0.8 mm) was designed to be narrower than the width of the outer medium channels (3.0 mm) that supply the bulk of the culture medium (fig. S1A). Arrays of polydimethylsiloxane (PDMS) pillars alongside the channel edges separate adjacent channels and prevent the leakage of prepolymerized hydrogel upon injection (fig. S1A). Similarly, the three-channel device consists of three adjacent, parallel channels having rectangular cross sections with dimensions shown in Fig. 2A. For simplicity and ease of fabrication, in the three-channel device, we replaced the pillar arrays with continuous PDMS lips to separate the middle channel from the side channels and thus contain the injected cell-embedded prepolymerized fibrin gel.

Acrylic molds were fabricated by cutting (Epilog laser cutter) our designs through 1-mm-thick acrylic sheets (fig. S1B). In three-channel devices, 0.3-mm-deep grooves were etched on the top surface of the acrylic sheet, while for seven-channel devices, rows of holes were fully cut through. Next, the laser-cut acrylic geometries were bonded to larger acrylic bases by applying 30-psi pressure at 85°C for half an hour (fig. S1B). These molds were then taped to the bottom of 60-mm Petri dishes to fabricate the final PDMS devices, as described previously (*65*, *66*). Briefly, PDMS and cross-linker (10:1; SYLGARD 184; Dow Corning) were mixed, degassed, and poured over the mold to a height of 6 mm. After curing for 4 hours at 60°C, the PDMS was cut and peeled from the mold. Gel filling ports and medium access ports were created by punching out 1- and 4-mm holes, respectively, using a biopsy punch (Miltex). The device was cleaned with tape to remove debris and then sterilized in an autoclave. Last, the bottom of the device and a glass coverslip (No. 1; Electron Microscopy Sciences) were treated with plasma for 60 s and pressed firmly together to form an irreversible bond. To restore hydrophobicity of surfaces and create irreversible bonding, the final devices were placed inside an 80°C oven overnight.

### Cell culture and device seeding

HUVECs and normal human lung fibroblasts (Lonza) were passaged and cultured in EGM-2MV and FGM-2 media (Lonza), respectively. Cells were initially expanded and cryopreserved to establish a consistent stock for all experiments. For an individual experiment, cells were plated onto collagen-coated flasks and grown to confluency before seeding into the fluidic devices. Except for conditioned medium experiments, EGM-2MV medium was used exclusively after seeding into fluidic devices. To fluorescently label the cells for live imaging, ECs and fibroblasts were stably transduced to express nonlocalized green fluorescent protein (GFP) and mCherry, respectively.

Before seeding into the devices, ECs and fibroblasts were resuspended at 16 million and 8 million cells/ml, respectively, in EGM-2MV supplemented with bovine thrombin (4 U/ml; Sigma-Aldrich). For JCC, equal volumes of the two cell suspensions were combined and gently mixed, except for experiments that investigated the effects of [Fig F1]cell density (fig. S4, E to G). For each device, 50 μl of the combined cell suspension was added to 50 μl of bovine fibrinogen [6 mg/ml in phosphate-buffered saline (PBS) without calcium or magnesium; Sigma-Aldrich] to form a final encapsulation suspension of ECs (4 million cells/ml) and fibroblasts (2 million cells/ml) in fibrinogen (3 mg/ml) with thrombin (2 U/ml). The suspension was mixed slowly several times over ice to avoid premature polymerization and then pipetted into the vascularization channel, i.e., half the volume through each of the two gel filling ports. The device was immediately placed in a humidified chamber and incubated at room temperature for 30 min during fibrin polymerization. Last, the medium channels were filled with warm EGM-2MV through the medium access ports. The devices were incubated at 37°C and 5% CO_2_ for the remainder of the culture, and the growth medium in the medium channels was replaced every day.

### Chemical inhibitor treatments

Chemical inhibitors were reconstituted in dimethyl sulfoxide (maximum concentration of 10 μl/ml that showed no effects on networks; VWR) and diluted to the effective concentrations recommended by the supplier and the literature, i.e., verteporfin (YAP inhibitor, 0.25 μM; Sigma-Aldrich). Cells were encapsulated in the JCC system and exposed to these concentrations of chemical inhibitors for up to 1 week (fig. S7). Cell viability was evaluated using a cell counting machine (NC-3000; ChemoMetec, Denmark).

### siRNA transfections

YAP siRNA was purchased from Dharmacon (USA) with the stock concentration of 20 μM. Fibroblasts were cultured in a six-well plate (1 × 10^5^ per well) and grown to 50% confluency. The medium in each well was replaced with 1.2 ml of serum-free medium containing the delivery reagent (Fisher Scientific Ltd.) and siRNA in a final concentration of 50 nM. After 6 hours, 1 ml of FGM-2 medium was added to each well, and the cells were kept in an incubator for 72 hours without replacing the medium. Following this, the transfected fibroblasts were exposed to fresh medium and were seeded in the devices following the JCC system protocol. The same procedure was followed for scrambled siRNA–treated fibroblasts.

Transfection efficiencies were examined by conducting Western blots on cells at both the 3-day and 7-day post–siRNA treatment time points (Fig. 4B; all materials were obtained from Bio-Rad Laboratories Ltd., unless otherwise stated). Total cellular protein content was isolated from lysed cells and separated by mass using SDS–polyacrylamide gel electrophoresis. A 10% precast polyacrylamide gel was used to resolve YAP proteins. The separated proteins were wet-transferred to a nitrocellulose membrane and subsequently incubated with the following primary antibodies, YAP (1:500 dilution; Insight Biotechnology, USA) and β-actin (1:3000 dilution; Sigma-Aldrich, USA). Following 24 hours of incubation, the primary antibodies were washed, and horseradish peroxidase (HRP)–conjugated secondary antibodies (Thermo Fisher Scientific, USA) were applied to the nitrocellulose membrane in a 1:3000 dilution. The secondary antibodies were washed, and the membrane was exposed to HRP developing solution (Bio-Rad, USA). Imaging was conducted using an Amersham Imager 680.

### Network length and lumen diameter

3D confocal images (21 slices, 10-μm *z*-step) of GFP-ECs were acquired with a 10× objective [numerical aperture (NA) 0.3; Olympus] on days 1, 4, 7, 10, and 14 after culture to quantify network length, diameter, perfusability, permeability, and barrier function. For each device, at least 10 randomly selected regions (each 1.27 mm by 1.27 mm) were imaged. Measurements were averaged together to obtain a sample value for each device that was used for statistical analysis. To quantify characteristic parameters of the network, stacks were projected using the “Maximum Intensity Z Projection” plugin in ImageJ. For network length, processing, binarizing, and skeletonizing of the images were performed using ImageJ. Using the “Analyze Skeleton” plugin, the length of all connected networks was calculated. The longest connected network was used to determine network length. To calculate 3D vessel diameters, the Filament Tracer extension for Imaris (Bitplane) was used to analyze the binarized, 3D reconstructed images (*67*). Lateral and transversal diameters at any point were determined from the cross section of the binarized vessel perpendicular to the skeleton axis at that point.

### Perfusability, permeability, and barrier function

A fluorescent tracer (70-kDa dextran; 0.25 mg/ml in EGM-2MV; Life Technologies) was used to assess functionality of the vasculature, including perfusability, permeability, and barrier function. Perfusability was calculated as the percentage of perfusable vessel structures. To measure perfusability, the downstream and upstream ports were half and fully emptied, respectively (fig. S1E, a and b), and then, the fluorescent tracer was introduced into the upstream port (fig. S1E, c). This created a hydrostatic pressure of ~30 Pa, thus flowing the tracer into the vascularized channel (fig. S1E, d). Images were acquired using a confocal microscope (Olympus, FV-1000) with 10× objective and *z*-stacks at 10 μm per slice. The stacks from both GFP-ECs and tracer channels were resliced and binarized. The EC channel was used to identify vessel outlines. For all *x*-*z* cross sections, if a vessel cross section contained dextran, then its area was added to the total perfused area. Perfusability was calculated by dividing the total perfused volume by the total vascular volume.

Permeability was measured following a previously reported protocol (*33*). Briefly, a fluorescent tracer was introduced into the vasculature, following the method described above for perfusability measurements. The vascularization channel was imaged immediately after introducing the tracer and again after 20 min. The average tracer intensity in both the vascular and interstitial spaces was calculated at both time points. The difference in interstitial intensity along with morphological properties of the vasculature was used to calculate the permeability coefficient as described in (*33*).

To account for conditions in which permeability could not be meaningfully calculated, i.e., where vascular lumens were not well defined or perfusion was highly nonuniform, we defined the “barrier function” parameter (Fig. 1E). To calculate barrier function, fluorescent tracer was introduced into the vasculature and imaged, following the method described above for perfusability measurements. A *z*-projected stack of the endothelium (GFP-ECs) was used to define the vascular and interstitial regions. Barrier function was calculated as the inverse of average intensity of fluorescent dextran in the interstitial region.

### Atomic force microscopy

The tissue was cut out gently from the fluidic device through the PDMS using a scalpel (Bard-Parker) along the perimeter of the vascularization channel (fig. S2F, a and b). For AFM measurements, the tissue was left attached to the PDMS (fig. S2F, c) which provided a supportive base and helped maintain the original tissue geometry. The entire sample was submerged in a CO_2_-independent buffered medium, consisting of Leibovitz’s L-15 Medium without phenol red (Gibco Life Technologies), and supplemented with 10% fetal bovine serum (FBS; Invitrogen). AFM force-distance measurements were acquired using a JPK NanoWizard CellHesion 200 (JPK Instruments AG, Berlin, Germany) placed on an inverted optical microscope (Zeiss Axiovert 200) with manual *xy*-positioning stage. Spring constants of the cantilevers were determined using the thermal noise method of the AFM software (JPK SPM, JPK instruments). Tipless cantilevers (nominal spring constant of 0.07 N/m; MLCT-O10, Bruker) were modified by gluing 50-μm-diameter glass microspheres (Cospheric, USA) to the tip of the cantilever via ultraviolet curing glue (ultraviolet curing, Loctite). The stage was carefully moved to position the cantilever tip above the middle of the sample before approaching the tissue surface. No less than 50 force-distance curves were taken per sample by moving the stage manually (in ~0.5-mm steps) across the length of the tissue, with an approach speed of 10 μm/s and a set force of 30 nN. Using a custom-written MATLAB (MathWorks) routine, the apparent elastic modulus *E*/(1 − ν^2^) was extracted by fitting the force-indentation curve to a Hertz contact model between a sphere and an infinite half space. After performing the initial stiffness measurements on the tissue, the tissues were incubated with cytochalasin D (4 μM; Sigma-Aldrich) for 15 min to inhibit active force generation through the inhibition of actin polymerization, and AFM measurements were repeated. Similar AFM indentation tests were performed on the tissues that were decellularized and resubmerged in Leibovitz’s L-15 + FBS medium.

Contributions to tissue stiffness as reported in Fig. 3 (D to F) were calculated using the following expressions

$$\text{Contribution of base fibrin gel}\;(\%)=100\times \frac{E_{\mathrm{fibrin}}}{E_{\mathrm{CNT}}}$$

where *E*_fibrin_ and *E*_CNT_ represent the elastic modulus of base fibrin gel and the control JCC system, respectively.

$$\text{Contribution of ECM remodeling}\;(\%)=100\times \left(\frac{E_{\mathrm{Decellular}}-E_{\mathrm{fibrin}}}{E_{\mathrm{CNT}}}\right)$$

where *E*_Decellular_ represents the elastic modulus of the tissue after decellularization.

$$\text{Contribution of cell material}\;(\%)=100\times \left(\frac{E_{\mathrm{CytoD}}-E_{\mathrm{Decellular}}}{E_{\mathrm{CNT}}}\right)$$

where *E*_CytoD_ represents the elastic modulus of the tissue after cytochalasin D treatment.

$$\text{Contribution of active force}\;(\%)=100-\text{contribution of other factors}=100\times \left(\frac{E_{\mathrm{CNT}}-E_{\mathrm{CytoD}}}{E_{\mathrm{CNT}}}\right)$$

For retinal stiffness measurements, eyes were enucleated from P3 or P7 mouse pups followed by retina dissection in PBS. All animal procedures were performed in accordance with the institutional Animal Welfare Ethical Review Body and U.K. Home Office guidelines on mixed-background mice (C57Bl6/J;129/Sv). Following dissection, each retinal tissue was transferred to Cell-Tak–treated (Cell and Tissue adhesive, BD Biosciences) glass-bottom Petri dishes. The semiwet tissue was left in the dish for 2 min to adhere to the glass and then Leibovitz’s L-15 + FBS medium was added for AFM measurements. AFM indentation tests were conducted on five radial regions approximately 400 μm apart. Following initial AFM measurements, the medium was replaced with cytochalasin D–supplemented medium, and the AFM tests were repeated to estimate the contribution of active contractile forces.

### Traction force microscopy

To estimate the mechanical stresses applied by cells onto the fibrin gel, a 3D traction force microscopy experimental pipeline was used. First, fibrin gels were fluorescently labeled with a far-red dye (Alexa Fluor 647 NHS succinimidyl ester, Thermo Fisher Scientific) that binds to free amine groups and has no known mechanobiological effects on the functionality of fibrin monomers (*45*). This allowed us to measure the gel at baseline levels (no cells) and also to visualize cell-driven gel displacements after the addition of cells to the fibrin gel. For each time point (days 1, 4, 7, and 10), cultured microfluidic devices were mounted onto the environmental chamber stage of an Olympus IX81 confocal microscope and imaged using a 20× objective (NA 0.4; Olympus). 3D *z*-stacks were captured before (stressed configuration) and after 15 min of treatment with the actin inhibitor, cytochalasin D. Cytochalasin D was added through the medium channel, and care was taken not to disturb the device. Fluorescent images of the cells and the fibrin gel were acquired before and after cytochalasin D treatment. Subsequently, Triton X-100 was added, and *z*-stack images of the final decellularized fluorescent gel were acquired. These sequential stacks were cross-correlated using an open source computational algorithm called fast iterative digital volume correlation (FIDVC) (*68*, *69*). A series of MATLAB (MathWorks) routines were written or modified from the open repository of Franck Lab (https://github.com/FranckLab) to postprocess the displacement from FIDVC and to obtain (i) displacement histograms (Fig. 3J) and (ii) a binarized stack from the fluorescence images of both endothelial and stromal cells in the stressed configuration (Fig. 3G) and (iii) to calculate the strains (Fig. 3H) (*70*, *71*).

### RNA sequencing

To isolate cells for RNA sequencing, the extracted tissue was dissociated with 2.5% trypsin (Gibco) for 1 hour in an incubator with occasional mixing. The trypsin was then diluted 1:50, and the cells were passed through a 35-μm strainer in preparation for fluorescence-activated cell sorting with a BD FACSAria II cell sorter to separate GFP ECs and nonfluorescent fibroblasts. Sorted ECs and fibroblasts were independently pelleted and resuspended in 1 ml of TRIzol (Invitrogen), and the manufacturer’s instructions (Invitrogen) were followed for RNA isolation. For sequencing, 3′ digital gene expression libraries were prepared on the basis of a previously published protocol (*72*) .

After sequencing, quality control on each of the libraries was performed to assess coverage depth, enrichment for mRNA (exon/intron and exon/intergenic density ratios), fraction of rRNA reads, and number of detected genes using bespoke scripts. Sequences were aligned against the human genome hg19 using bwa (*73*). Gene expression was estimated on the basis of reads mapping near the 3′ end of transcripts using ESAT (*74*) based on the Refseq annotation. Results were summarized as counts per million mapped reads, merged across samples, log-transformed, and subjected to hierarchical clustering and visualization. Differential expression analysis was performed in the R v. 3.2.3 statistical environment using the DESeq2 package (*75*). Differences in gene expression between conditions (expressed as log_2_-transformed fold changes in expression levels) were estimated under a general linear model framework fitted on the read counts, modeled under a negative binomial distribution. Differential expression significance was assessed using a Wald test on the fitted count data. Independent filtering based on the mean of normalized count of the differentially expressed genes was turned off, as well as Cook’s distance-based filtering. *P* values were adjusted for multiple comparisons testing using the Benjamini-Hochberg. Functional annotation was performed using DAVID v6.8 (*76*, *77*). Principal components were extracted from log-regularized count data using DESeq2 (fig. S6, A and B).

### Gene set enrichment analyses

Sequencing data were processed and analyzed using the GSEA software, developed by the Broad Institute of MIT and Harvard (USA) and available at www.broadinstitute.org. For analyses of EC and fibroblast transcriptome by GSEA (fig. S6, C and D), gene count RNA sequencing data were first preprocessed using the Voom Normalization from the GenePattern platform developed by the Broad Institute of MIT and Harvard (USA) and available at GenePattern (www.genepattern.org), following the program’s guidelines (expression value filter threshold = 1). This module preprocesses RNA sequencing data into a form suitable for use downstream in analyses such as GSEA. Because of poor quality (low RNA counts), sample CH4_B (HUVECs in coculture, day 4, sample B) was excluded from subsequent analyses. Preprocessed data were then run through GSEA software following the program guidelines. The specific settings applied in all analyses are as follows: Number of Permutations (1000), Permutation Type (Gene set), Enrichment statistic (Weighted), and Metric for ranking genes (*t* Test).

### Fluorescent staining

To stain the JCC system with VE-cadherin and YAP antibodies (fig. S8), the intact tissue construct was extracted from the device as described above. The tissue was then fixed with 4% paraformaldehyde for 15 min at room temperature, followed by three washes with PBS. To permeabilize the cells, the tissue was treated with 0.3% Triton X-100 for 10 min. The tissue was blocked with 3% BSA (bovine serum albumin), 5% normal goat serum, and 0.1% Triton X-100 solution for 1 hour. YAP (1:100; sc-101199, Santa Cruz Biotechnology) and VE-cadherin (1:100; 2500S, Cell Signaling Technology) primary antibodies diluted in blocking solution were introduced to stain the cells in the tissue construct for 3 hours. The tissue was thoroughly washed three times with PBS, followed by the addition of subsequent secondary antibodies for 2 hours. The tissue was washed and stored in PBS at 4°C. The fluorescently labeled tissues were imaged using an upright confocal microscope (Zeiss) with 25× water objective. Tissues from three different devices were imaged for each condition, and in each tissue, three randomly selected regions of interest were imaged. To analyze the data, each image was projected using the “sum slices” method in ImageJ, and the intensity inside the cells was calculated using the “Freehand Selections” tool. For each image, at least 100 cells were analyzed.

To visualize cell nuclei, ECs junctions, and cellular membranes on day 7 (fig. S2B), the JCC tissue constructs were incubated for 5 min with Hoechst (New England Biolabs), anti-human CD31 Alexa Fluor 647 (BD Biosciences) and CellMask plasma membrane stain (Thermo Fisher Scientific), respectively. The fluorescently stained vasculature was then imaged by confocal microscopy (Olympus) using 10×, 20×, and 60× objectives.

To stain mouse retina (fig. S5A), eyes were first enucleated from P3 or P7 mouse pups and then fixed in 4% formaldehyde in PBS for 10 min. The retina was then dissected and further fixed using methanol. Whole-mount fluorescent labeling of ECs was performed using a biotinylated anti-IB4 antibody (L2140, lot 085M4032V, Sigma-Aldrich) followed by Alexa Fluor 488–conjugated streptavidin (Thermo Fisher Scientific), as described previously (*78*).

### Quantification and statistical analysis

Sample size was determined by following published studies, and therefore, no statistical methods were used to predetermine sample size. The experiments were not randomized, and because of their nature, the investigators were not blinded to allocation during experiments or analysis of results. At least three devices from two or three independent experimental repeats were analyzed for quantification and statistical comparison. Quantification, statistical analysis, and plotting were performed in MATLAB (MathWorks) or Origin (OriginLab). In notched box plots, the notch represents median; the filled square, mean; the top and bottom of the box, 75th and 25th percentiles, respectively; and whiskers, SD. The overlaid data points represent the experimental data binned into about 10 to 20 equally spaced intervals. In bar plots, the bar denotes mean and the error bar denotes either SE or SD. Normality of distributions was tested using the Lilliefors test. For normal distributions, the difference between groups was evaluated by either two-tailed Student’s *t* test (for two groups) or linear analysis of variance (ANOVA) followed by Tukey-Kramer test to obtain the multiple comparisons two-sided *P* values. For non-normal distributions, either two-sided Mann-Whitney *U* test (for two groups) or Kruskal-Wallis test followed by Dunn’s multiple comparisons post-test was applied to compare the groups and obtain two-sided *P* values. *P* values of <0.01 were considered statistically significant (*P < 0.01, **P < 0.001, and ***P < 0.0001), while “ns” indicates no statistically significant difference.

### Mouse strains

All animal experiments were carried out in accordance with the U.K. Animal (Scientific Procedures) Act 1986 and U.K. Home Office regulations under project license number PP0736231. Mice carrying the conditional floxed alleles Yap1<tm1c(KOMP)Mbp> (Yap1^fl/fl^; cKO) were a gift from H. Gerhardt and A. Behrens. C57BL/6-Tg(Pdgfra-cre)1Clc/J mice were acquired from the Jackson Laboratory (stock no. 013148). Gt(ROSA)26Sor^tm4(ACTB-tdTomato,-EGFP)Luo^ (R26-mTmG) mice were supplied by the Francis Crick Institute BRF core colony.

### Embryo isolation and preparation

Embryos were generated in timed matings by breeding Yap1^fl/fl^ females with Yap1^fl/+^; PDGFRα Cre-positive males. Embryos were dissected at embryonic stages E10.5, E11.5, and E12.5. Embryos were transferred to cold PBS (pH 7.4) for isolation under a dissecting microscope. Embryos were then fixed in either 10% neutral-buffered formaldehyde or 4% paraformaldehyde overnight and then stored in either 70% ethanol (for formalin-fixed paraffin-embedded (FFPE) sections) or PBS (for whole-mount immunofluorescence). Macroscopic pictures of mouse embryos at E10.5, E11.5, and E12.5 were taken after fixation (Fig. 5A).

### Immunohistochemistry and immunofluorescence on mouse tissue

Embryos fixed in 10% neutral-buffered formaldehyde were paraffin-embedded, sectioned at 4-μm thickness, and stained with hematoxylin and eosin (Fig. 5B) or standard immunohistochemistry protocols by the Experimental Histology Laboratory at the Francis Crick Institute. For immunohistochemistry anti-YAP1 primary antibody was used (1:200; Santa Cruz Biotechnology, sc-4912) diluted in signal stain (Cell Signaling Technology, #8112) with pH 6 citrate buffer antigen retrieval (fig. S9D). For Immunofluorescence on FFPE sections (Fig. 5C and fig. S9E), tissue was deparaffinized and rehydrated. Antigen retrieval was performed using Antigen Unmasking Solution high pH (Vector Laboratories, #3301) in the microwave, 2 min at 800-W power and 18 min at 450 W. Sections were incubated for 1 hour at room temperature in blocking buffer (2% BSA and 10% normal donkey serum in PBS-Tween 0.1%), followed by an overnight incubation at 4°C with primary antibody in staining buffer (1% BSA and 5% normal donkey serum in PBS-Tween 0.1%). Primary antibodies used for immunofluorescence on FFPE were anti-PDGFRα (1:50; R&D Systems, AF1062), anti-GFP (1:200; Abcam, ab13970), anti-endomucin (1:100; Santa Cruz Biotechnology, sc-65495), anti-NG2 (1:100; Millipore, AB5320), and anti-desmin (1:75; Dako, M0760). Sections were washed 3 × 5 min in PBS-Tween 0.1%, followed by incubation with secondary antibodies (Alexa Fluor–conjugated; Thermo Fisher Scientific, A31573, A21082, A31571, and A48270 and Jackson ImmunoResearch Laboratories, 703-545-155) 1:1000 in staining buffer for 45 min at room temperature. Slides were incubated with 4′,6-diamidino-2-phenylindole (1 mg/ml; 1:1000 in PBS; Sigma-Aldrich, d9542) for 10 min and washed with PBS afterward. Slides were mounted with ProLong Diamond Antifade Mountant (Thermo Fisher Scientific, P36965). Images were acquired on the Zeiss 780 and 880 confocal microscopes.

### Whole-mount immunofluorescence

Embryos were permeabilized with 0.5% Triton X-100 PBS for 1 hour at room temperature on a rocking platform. Blocking followed using PBSMT [PBS (pH 7.4) with 3% milk and 0.1% Triton X-100] two times for 1 hour at 4°C on a rocking platform. Embryos were incubated with primary antibody against endomucin (1:100; Santa Cruz Biotechnology, sc-65495) for 72 hours at 4°C on a rocking platform. Embryos were then washed five times for 1 hour in PBMST at 4°C on a rocking platform. Embryos were incubated with secondary antibody Alexa Fluor goat–anti-rat secondary antibody 647 (Thermo Fisher Scientific, A21247), 1:500 in PBSMT for 72 hours at 4°C on a rocking platform protected from light. Embryos were then washed in PBSMT five times for 1 hour, followed by two washes for 1 hour each in 0.1% Triton X-100 in PBS at 4°C on a rocking platform protected from light. Embryos were then incubated with increasing glycerol concentrations for 30 min each (10, 20, 40, and 60% glycerol in PBS) and lastly stored protected from light in 80% glycerol in PBS at 4°C until imaging on a Zeiss 780 confocal microscope using a glass bottomed cell culture plate (MatTek; Fig. 5D and fig. S9B). Colocalization analysis was performed with the ZEN black software (fig. S9C).

### Vessel segmentation

Blood vessels were segmented from whole-mount immunofluorescence images of E10.5 embryos using a combination of deep learning and manual annotation. Briefly, a deep convolutional neural network, with U-net architecture (*79*), was pretrained using 3000 3D simulated blood vessel networks with predefined range of branching properties (radius, angle, tortuosity, etc.) representative of normal tissue. Input volumes were 64 × 64 × 64 voxels in size. Encoding blocks in the neural network contained two 3D convolutional layers and a max-pooling layer, with ReLu activations. Decoding blocks contained convolutional layers, skip connections to the corresponding encoding blocks, and ReLu activations other than at the output layer, where sigmoid activations were implemented. All deep learning was undertaken with TensorFlow v2.3.0 in Python 3.7.

Five imaging volumes (64 × 64 × 64) were manually segmented from the embryo data using Amira software (Thermo Fisher Scientific). These regions were then used to fine-tune the pretrained neural network and enable automated segmentation of the remaining data. Following training, the network achieved a dice coefficient of 0.98, which was assessed using a further set of manually segmented data. Individual segmentation results were also assessed by eye, and where necessary, automated segmentations were manually edited using Amira.

### Vascular geometric analysis

Segmented blood vessel masks were converted into graph format using a 3D skeletonization algorithm in Python 3.7 from the skimage package (v 0.18.1). Vascular network geometry was quantified from the vascular networks according to branching angle, vessel segment radius, and branching distance (Fig. 5E and fig. S9, H and I).

### Transmission electron microscopy

For electron microscopy (fig. S9F), perfusion-fixed E10.5 embryos were postfixed by immersion in 4% (v/v) formaldehyde (TAAB Laboratories Equipment Ltd, Aldermaston, UK) in 0.1 M phosphate buffer (PB; pH 7.4) at 4°C overnight and stored in 1% (v/v) formaldehyde (TAAB ) in 0.1 M PB (pH 7.4) at 4°C until further processing. The embryo heads were dissected and processed using a PELCO BioWave Pro+ microwave (Ted Pella Inc., Redding, CA, USA) following a protocol adapted from the National Center for Microscopy and Imaging Research protocol (*80*). Each step was performed in the BioWave, except for the PB and water wash steps, which consisted of two washes on the bench followed by two washes in the BioWave without vacuum (at 250 W for 40 s). All the chemical incubations were performed in the BioWave for 14 min under vacuum in 2-min cycles alternating with/without 100-W power. The SteadyTemp plate was set to 21°C unless otherwise stated. Briefly, the samples were fixed again in 2.5% (v/v) glutaraldehyde (TAAB)/4% (v/v) formaldehyde in 0.1 M PB and stained with 2% (v/v) osmium tetroxide (TAAB)/1.5% (v/v) potassium ferricyanide (Sigma-Aldrich). They were then incubated in 1% (w/v) thiocarbohydrazide (Sigma-Aldrich) with the SteadyTemp plate set to 40°C and further stained with 2% osmium tetroxide in ddH_2_O (w/v). The samples were then incubated in 1% aqueous uranyl acetate (Agar Scientific, Stansted, UK) with the SteadyTemp plate set to 40°C and washed in dH_2_O. The samples were then stained with Walton’s lead aspartate with SteadyTemp set to 50°C and dehydrated in a graded ethanol series (70, 90, and 100%, twice each) and 100% acetone (four times), at 250 W for 40 s without vacuum. Exchange into Durcupan ACM resin (Sigma-Aldrich) was performed in 50% resin in acetone, followed by four pure Durcupan steps, at 250 W for 3 min, with vacuum cycling (on/off at 30-s intervals), before embedding at 60°C for 48 hours in silicon molds. The blocks were then mounted for micro–computed tomography on a cylindrical specimen holder. Tomographic imaging was conducted in an Xradia Versa 510 (Carl Zeiss Ltd., Cambridge, UK). A low-resolution scan was captured at 40 kV/3 W with a 15-s exposure and with a pixel size of 3 μm. The data were exported as tiff, and the region of interest, on a transverse section of the upper part of the mesenchyme, was identified and targeted in each block using the Crosshair plugin in Fiji (*81*). Sections (70 nm) were cut using a Leica UC7 ultramicrotome (Leica Microsystems, Vienna, Austria) and picked up on Formvar-coated copper 1 mm–by–2 mm slot grids (Gilder Grids Ltd., Grantham, UK). For each sample, all the blood vessels visible in an ultrathin section were imaged using a 120-kV JEOL JEM-1400Flash Electron Microscope (JEOL Ltd., Welwyn Garden City, UK) and captured with JEOL’s Matataki Flash camera. For analysis, the interaction between mesenchymal cells and ECs was scored on a scale of 0 to 5 corresponding to No interaction, Very Limited, Limited, Fair, Fairly Extensive, and Extensive interaction (fig. S9G).

### AFM on embryo tissue

For embryo stiffness measurements (Fig. 5F), embryos at E11.5 were isolated and placed inside a six-well plate in cold PBS (pH 7.4). To keep the embryonic tissues fresh, the well plate was kept on ice for the ~5-hour duration of the AFM experiments. Each embryo was then transferred to a Petri dish and immobilized using a harp slice grid (ALA Scientific, NY, USA). The Petri dish was then placed on an AFM motorized stage interfaced with a top camera view. AFM indentation tests were conducted on a ~800 μm–by–~800 μm region above the eye and close to the edge of the head. At least 30 indention force-distance curves for each embryo were measured approximately 100 μm apart.

## Supplementary Materials

### This PDF file includes:

Figs. S1 to S10 Tables S1 to S3 Supplementary Notes ImageJ Macro Scripts
