# Supplementary material for: Emergent mechanical control of vascular morphogenesis
Source: Sci Adv. 2023 Aug 11;9(32):eadg9781. doi: 10.1126/sciadv.adg9781 (PMC10421067; doi:10.1126/sciadv.adg9781)
Supplement: Supplementary file 1 — Figs. S1 to S10 Tables S1 to S3 Supplementary Notes ImageJ Macro Scripts [file sciadv.adg9781_sm.pdf]

Science Advances
AAAS

Supplementary Materials for

Emergent mechanical control of vascular morphogenesis

Jordan Whisler *et al.*

Corresponding author: Erik Sahai, erik.sahai@crick.ac.uk; Roger Kamm, rdkamm@mit.edu; Emad Moeendarbary, e.moeendarbary@ucl.ac.uk

Sci. Adv. **9**, eadg9781 (2023)
DOI: 10.1126/sciadv.adg9781

This PDF file includes:

Figs. S1 to S10
Tables S1 to S3
Supplementary Notes
ImageJ Macro Scripts

Supplementary Figures

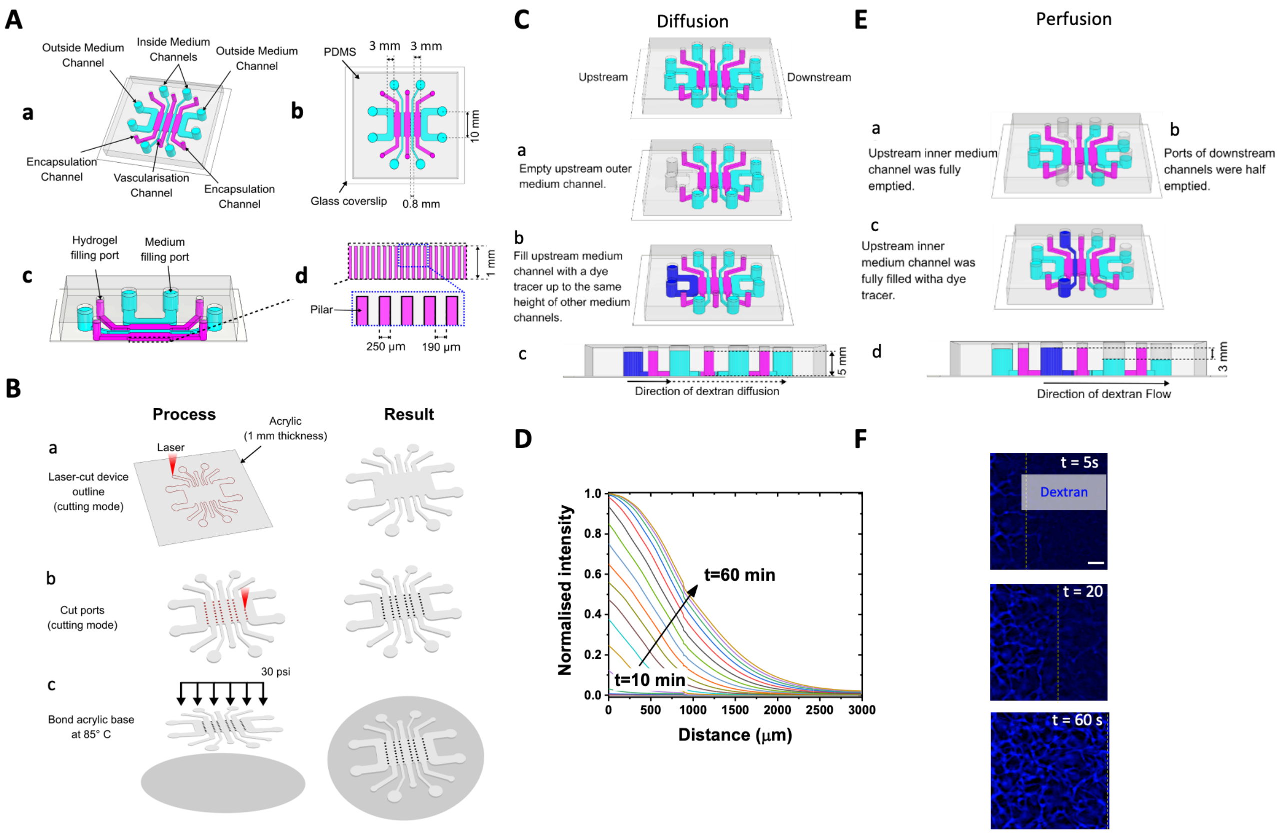

Figure S1: Microfluidic platform designs, fabrication, and handling. A, 7-channel device design. a) 3D view of the seven-channel device; two outer growth medium channels and two inner growth medium channels (in blue), as well as two lateral cell encapsulation channels and one central vascularization channel (in pink). b) 2D top view of the device showing the dimensions of the channels. A glass coverslip is bonded to the bottom of the PDMS device. The height of all channels is 1 mm. c) 1- and 4-mm ports enable injection of the hydrogel and growth medium into their respective channels. d) Array of PDMS pillars (height of 1 mm, diameter of 250 µm and edge to edge distance of 190 µm) lined along the channels to separate the individual channels and prevent leakage of the hydrogel after injection. **B,** Acrylic mold fabrication. 3-step process: a) Outline of the mold was cut out of 1-mm thick acrylic sheet; b) Array of holes were cut through at boundaries between adjacent channels; c) Mold was bonded to an acrylic base using force at high temperature to initiate melting and fusion. **C,** Diffusion assessment: a) Upstream outer medium channel was emptied and b) refilled with fluorescent tracer. c) No head pressure was applied to ensure pure diffusion of the tracer dye. **D,** Temporal profile of dextran diffusion throughout the middle gel channel. **E,** Perfusion assessment. a) Upstream inner medium channel was emptied. b) Ports of downstream perfusion channels were half emptied; c) Upstream inner medium channel was fully filled with the tracer dye. d) A hydrostatic pressure head of ~0.3 cm H_2O was applied across the central channel to initiate dextran perfusion through the central vascularized channel. **F,** Example of dextran perfusion in JCC system. Dashed line shows penetration of dextran into the tissue for various time points after its introduction into the side media perfusion channel at time t=0. Scale = 100 µm.

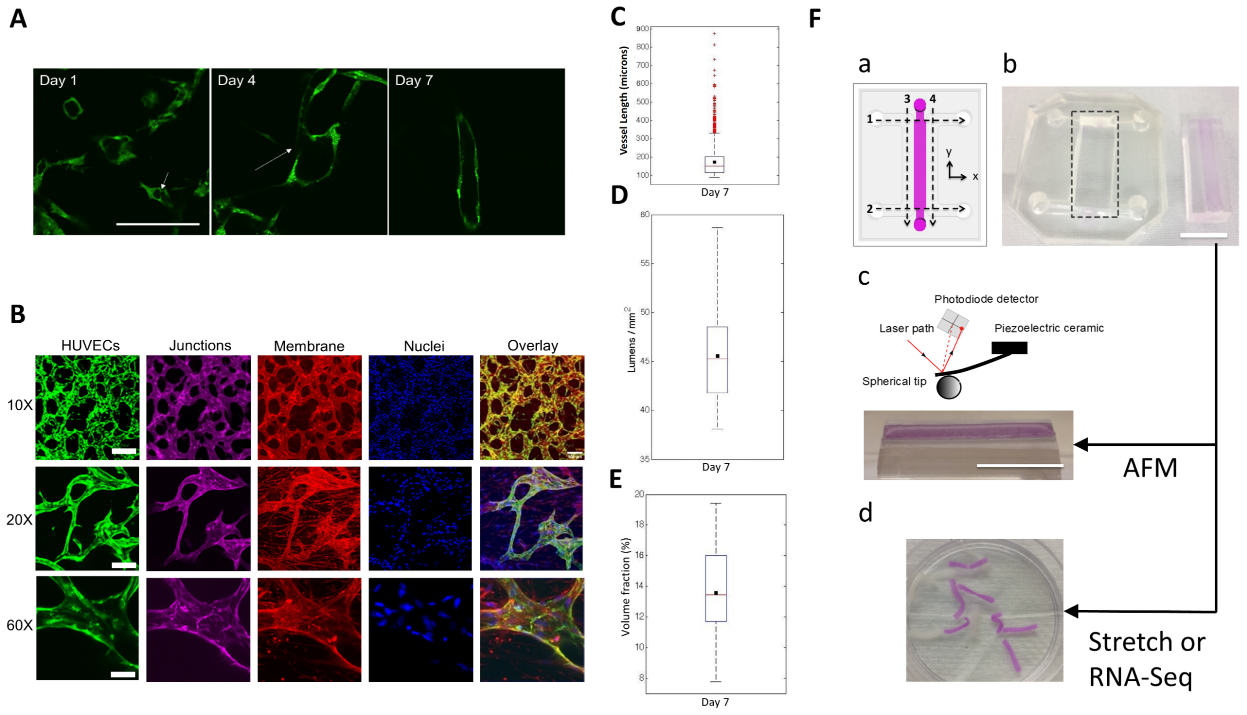

Figure S2: Imaging of cellular processes and tissue removal setup. A, EC lumen formation. Day 1: White arrow points to a single cell with multiple intracellular vacuoles. Day 4: White arrow points to the joining of intracellular vacuoles between two cells. Day 7: A single, multicellular vessel with well-defined lumen. Scale = 100 µm. **B,** Staining of the JCC system on day 7 for intercellular junctions (CD31, magenta), cell membrane (CellMask, red) and nuclei (Hoechst, blue). Scale = 150 µm, 150 µm, and 50 µm in the 10X, 20X and 60X images, respectively. **C-E,** Quantification of vessel length, vascular density and volume fraction at day 7. **F,** Tissue removal from the device for AFM, manual stretching and genomics measurements. a) Four cuts were made with a scalpel through the PDMS around the vascularization channel. Cuts were made along medium channels where PDMS was not bonded to the glass coverslip. b) Photo of the device after cutting and peeling vascularisation channel from glass coverslip. Scale = 1 cm. c) Tissue remained attached to PDMS which facilitated AFM indentation tests on the top surface. Scale = 1 cm. d) Complete detachment of the vascularised tissue prepared for manual stretching or genomics.

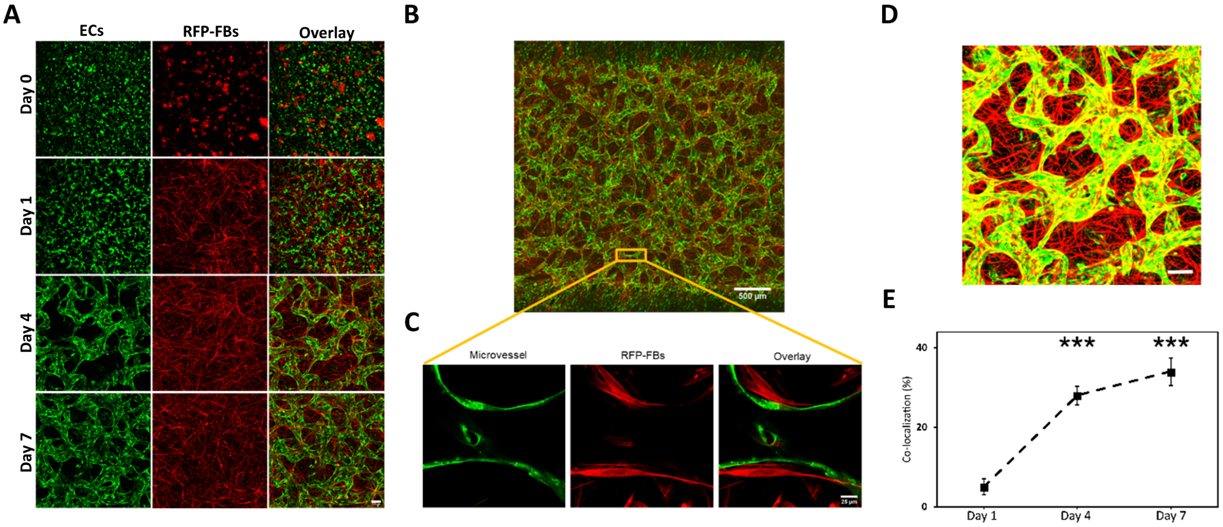

Figure S3: EC-fibroblast interactions. A, Progression of morphological changes in ECs and FBs during the course of one week. Scale = 100 µm. **B,** a large view of microvessels. Scale = 500 µm. **C,** High resolution, 3D confocal images showing FBs elongating along the microvessels. Scale = 25 µm. **D,** FB colocalization with microvessels indicated by yellow color levels. Scale = 150 µm. **E,** Colocalization of FBs with ECs measured over the course of one week. Colocalization percentage was calculated by finding Manders' coefficient in ImageJ JACoP plugin (***P value <.001, n=3).

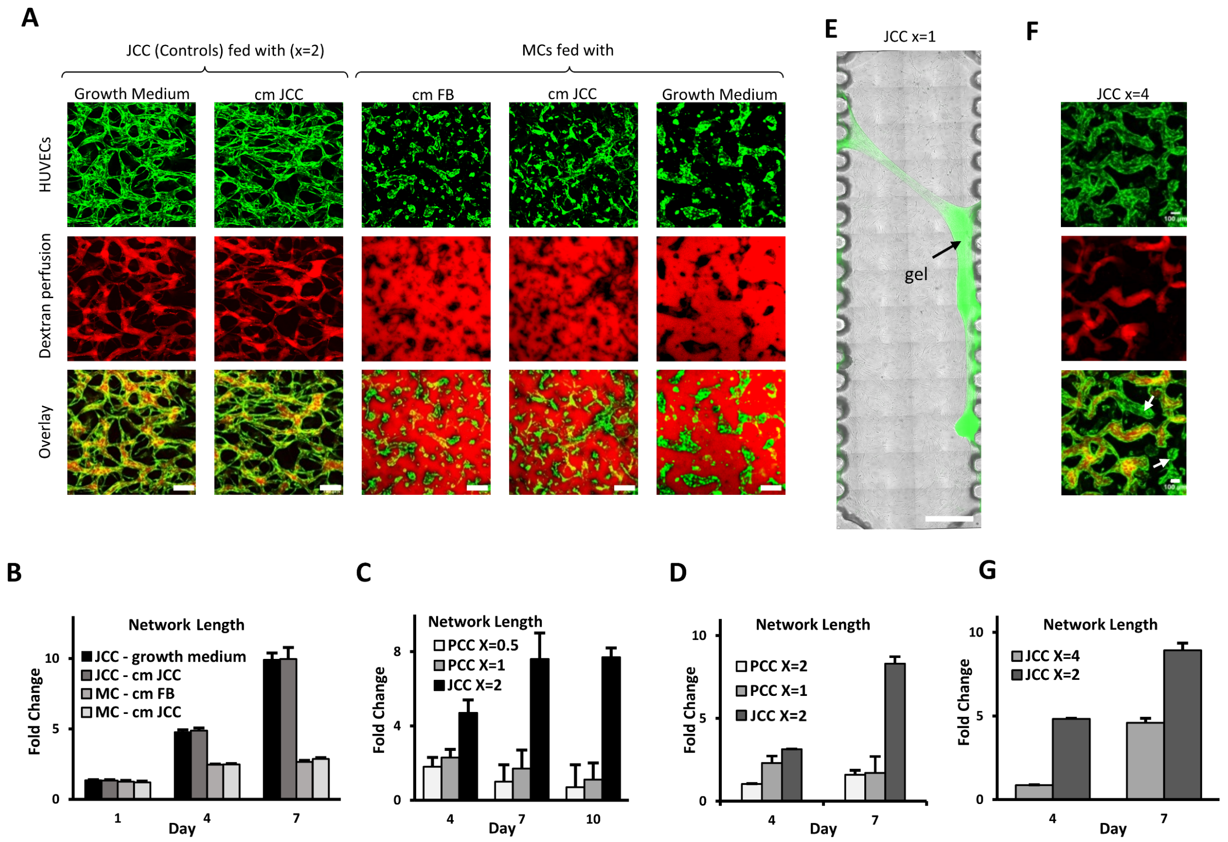

Figure S4: Impact of conditioned media and cell density. A, Comparison of EC morphology in response to different conditioned media. Confocal images of vascularized tissue constructs on day 7 showing the perfusability and the structure of vascular networks exposed to different conditioned media (cm JCC or cm FB). Except for the JCC controls, none of the other treatments resulted in perfusable vasculature. Scale = 100 µm. **B,** Assessment of the longest connected network length normalized to the day 1 MC value (i.e. ECs treated with growth medium). **C,** Effects of increasing FB seeding density in PCC system on the longest connected network length, normalized to the MC value on each day. In PCC X=1, 4 million cells /ml ECs were present in the central channel and 2 million cells /ml FBs in each of the side channels. In PCC X=0.5, 4M/ml ECs were seeded in the central channel and 4 M/ml FBs in each of the side channels. **D,** Effects of increasing EC seeding density in PCC system. In PCC X=2, 8 million cells /ml ECs were seeded in the central channel and 2 million cells /ml FBs in each of the side channels. **E,** Increasing the seeding density of FBs (4M /mL) led to gel contraction and deterioration. Scale = 1 mm. **F,** Reducing FB seeding density to 1M/ml (X= 4) led to a reduction in network perfuasiblity. Scale = 100 µm. **G,** Effect of FB seeding density on vascular network length in JCC system. X is the ratio of total number of ECs to the total number of FBs in the system. The standard JCC system had 4 million cells /ml ECs and 2 million cells /ml FBs in the vascularised channel and, therefore, X=2. In JCC X=4, 4 M/ml ECs and 1 M/ml FBs were seeded in the central vascularised channel.

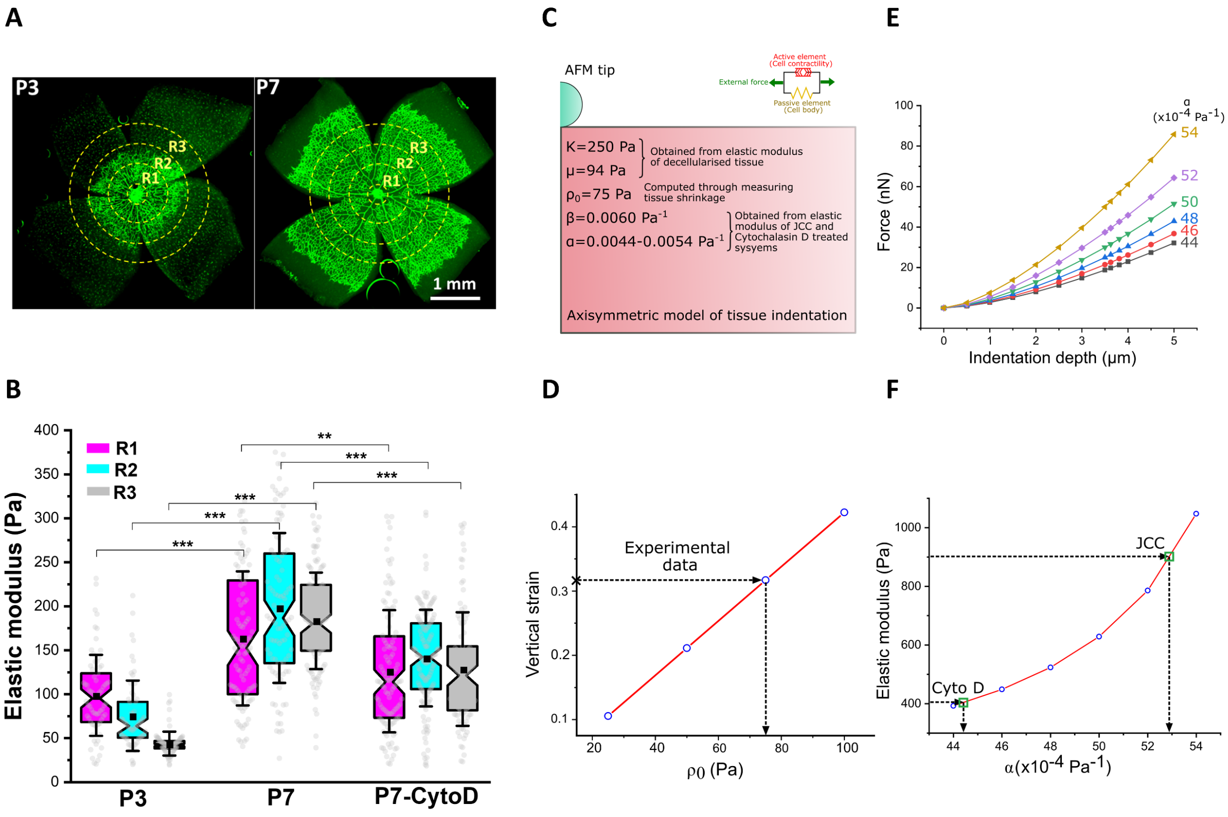

Figure S5: Active tissue stiffening due to cell generated forces. **A,** IB4 staining of P3 and P7 retinas. Scale = 1 mm. **B,** Stiffness of P3 and P7 mouse retinas measured at three increasing distances from the center of the retina. Effect of cytochalasin-D treatment on P7 retinas was also examined. Data for each specified region is from a minimum of 60 AFM indentation measurements on n=3 retinas. Statistical comparison was performed for each pair using two-sided Mann Whitney U-test. *P< 0.01, **P<0.001, ***P<0.0001. **C-F**, Computer simulations of indentation on active tissue to evaluate the impact of active forces on tissue elastic modulus. **C,** Geometry and parameters used in the finite element analysis of the indentation tests. An axisymmetric model of indentation of a material with an active-passive constitutive model was employed to predict the mechanical responses of the tissue. **D,** Calibration of ρ_0 by comparing simulations with experimental data of Fig. 3H for achieving similar levels of strain. **E,** Computational results for indentation of a tissue with different α parameter values. **F,** Effects of α parameter on active stiffness of tissue. Reducing α parameter value represents Cytochalasin D treatment which reduces active forces and consequently active stiffness.

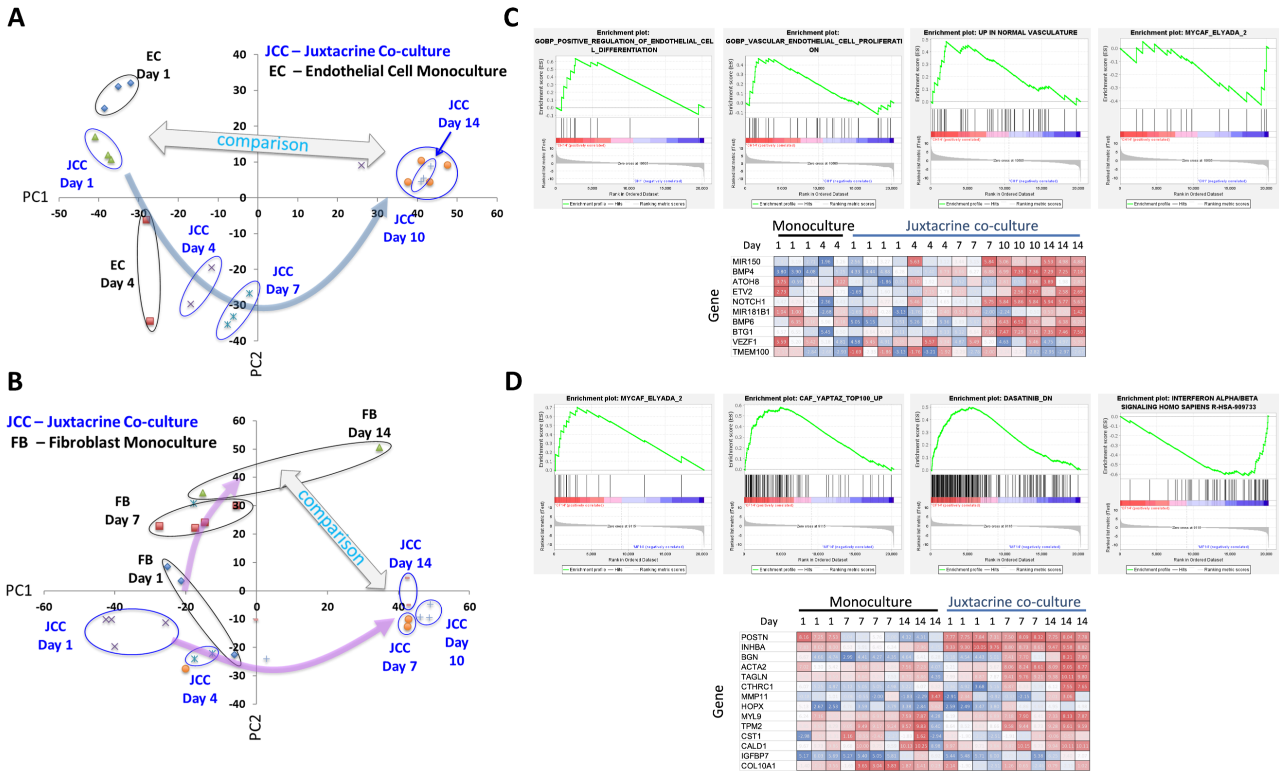

Figure S6: Transcriptomic analysis of EC – fibroblast crosstalk. A, Principal Component Analysis (PCA) plot shows the transition in the transcriptome of endothelial cells (ECs) when co-cultured with fibroblasts for increasing lengths of time. Blue arrow indicates the transition in transcriptional profile from day 1 to day 14. **B,** PCA plot shows the transition in the transcriptome of fibroblasts when co-cultured with EC for increasing lengths of time. Deep purple arrow indicates the transition in transcriptional profile from day 1 to day 14 in co-culture, lighter arrow indicates the transition in mono-culture. **C,** Plots show GeneSet Enrichment Analysis of genes associated with endothelial cell proliferation, differentiation, normal vasculature, and 'myoCAFs' comparing EC co-cultures on day 1 vs day 14 (indicated by grey arrow in (A)). Heatmap shows the expression of genes from the EC differentiation geneset in mono- and co-culture at indicated time (in days). **D,** Plots show GeneSet Enrichment Analysis of genes associated with 'myoCAFs', YAP-dependent transcription, Src-family kinase regulated genes, and interferon alpha target genes comparing fibroblast co-cultures on day 14 with the equivalent day 14 fibroblast mono-cultures (indicated by grey arrow in (B)). Heatmap shows the expression of genes from the 'myoCAF' geneset in mono- and co-culture at indicated time (in days).

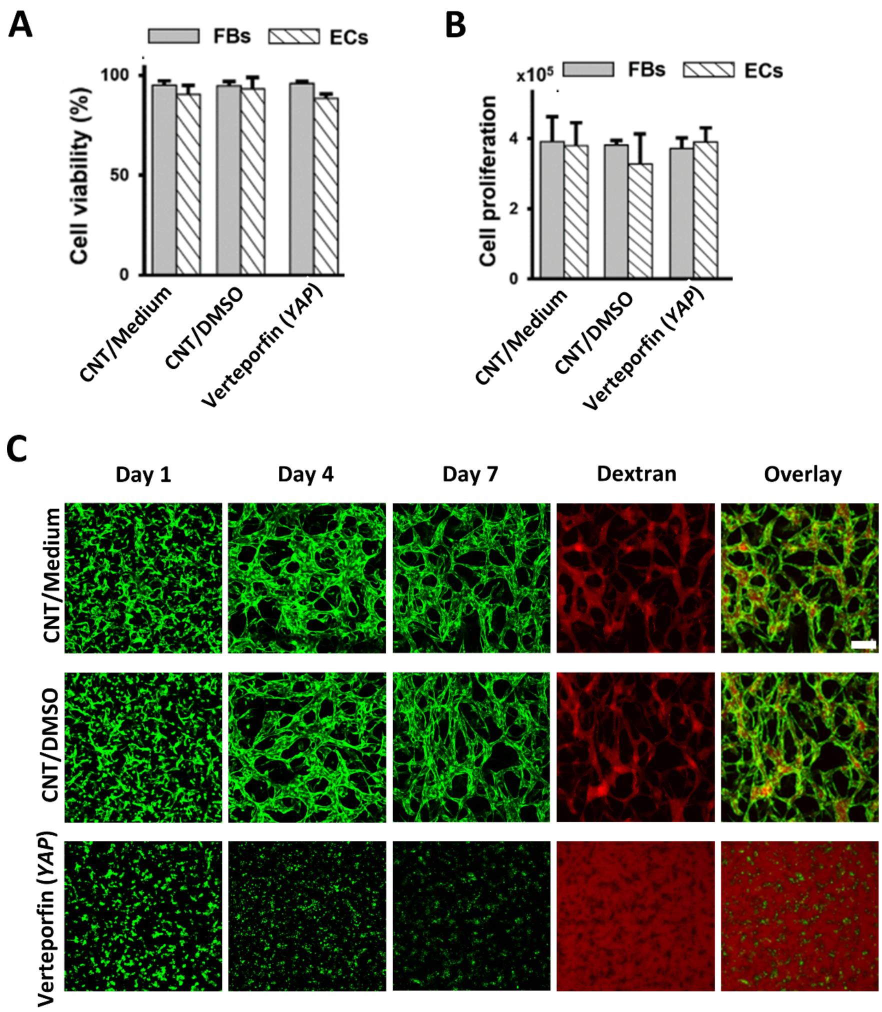

Figure S7: Perturbing mechanotransduction pathways in both ECs and fibroblasts using chemical inhibitors. A, B, Treatment of fibroblasts and ECs with verteporfin (YAP inhibitor – 0.25 µM) had no significant impact on cell viability shown in (A) and cell proliferation shown in (B). **C,** Confocal images of ECs (green) and perfused dextran (red) demonstrating the effects of inhibiting mechanotransduction genes (using drug treatments acting on both ECs and fibroblasts) on the JCC vascular network morphology and functionality on day 7. Scale = 150 µm. For drug treatments, a maximum DMSO concentration of 10 µl/ml was used which didn't affect the network perfusability and morphology. Network perfusability was fully impaired by inhibition of YAP.

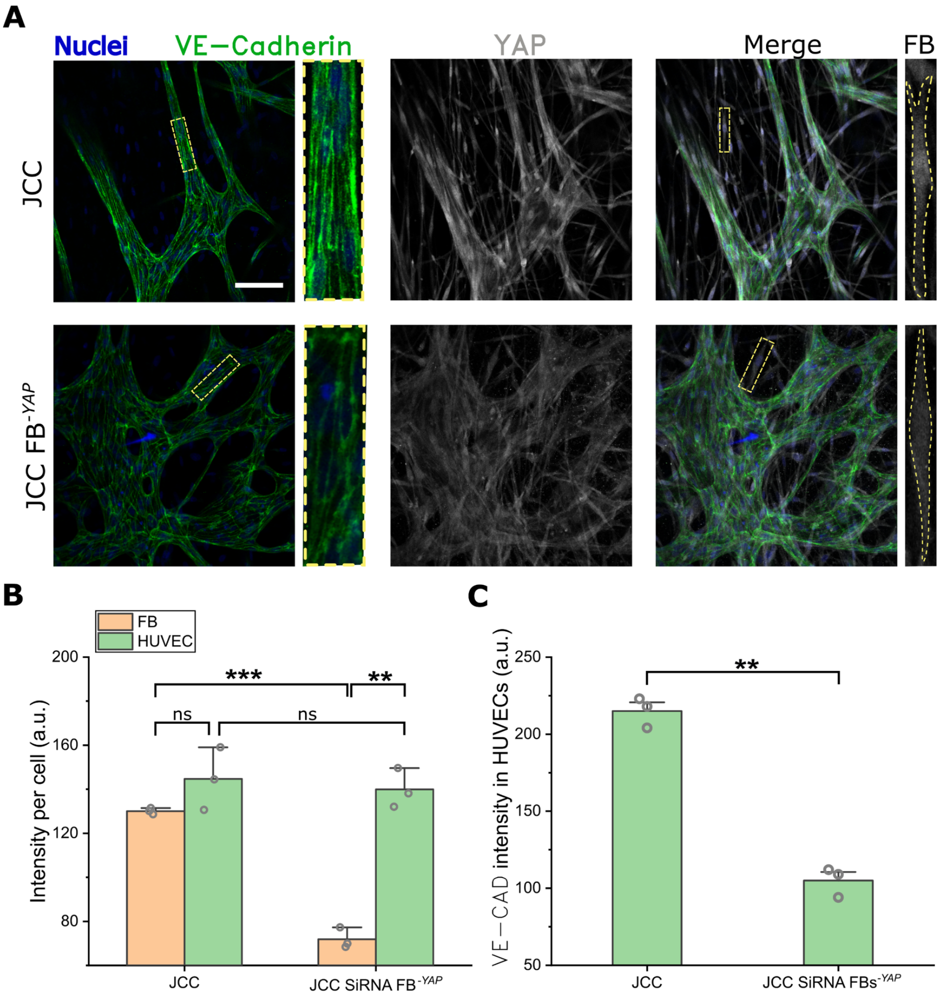

Figure S8: Impact of fibroblast YAP1 knockdown on expression of YAP and VE-Cadherin in JCC system. A, Immunofluorescence staining of VE-Cadherin (green), nuclei (blue) and YAP 1 (grey) in JCC and FB^{-YAP} JCC systems revealed that YAP knockdown in FBs was effective till day 7. Also, images show disturbance in VE-Cadherin due to FB^{-YAP} in JCC system. Insets show a zoom in view of VE-CAD in HUVECs and YAP in FB. Scale = 100 μm. **B,** Quantification of YAP average intensity in individual cells. **C,** Quantification of VE-Cadherin expression in HUVECs. (mean ±s.e.m., n=3 devices, ** *p*-value < 0.001 and *** *p*-value < 0.0001).

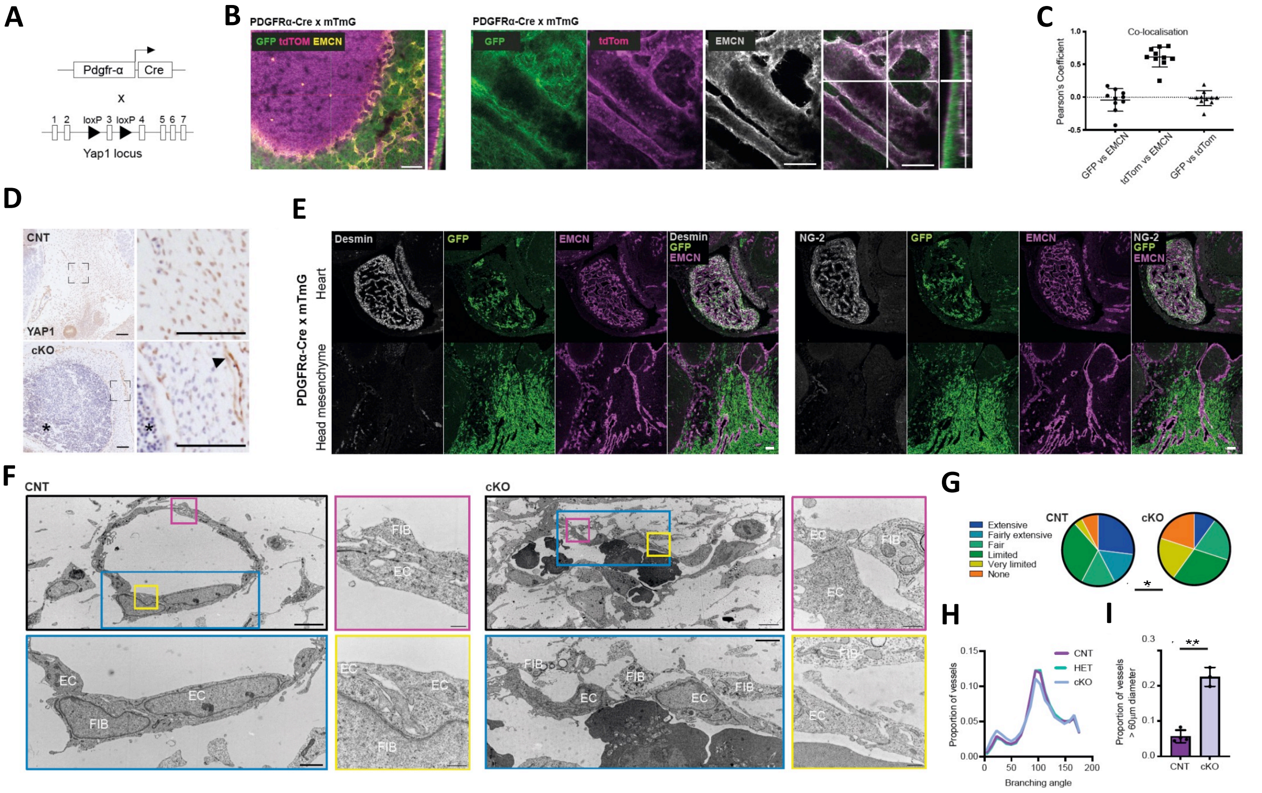

Figure S9: Loss of YAP1 in fibroblasts is embryonic lethal due to vascular defects. A, Schematic of the conditional YAP1 deletion (exon 3) by PDGFRa-driven Cre-recombinase expression. **B,** Wholemount analysis of endogenous fluorescence of PDGFRα-Cre+ x R26 mTmG (Cre+) embryos at E12.5. Left panels: Confocal image shows recombination in head mesenchyme (green) and not in brain tissue (magenta) upon PDGFRα-Cre expression. ECs stained for endomucin are shown in yellow. Scale = 500 µm. Right panels: Confocal image shows recombination in head mesenchyme (green) and not in ECs (magenta) upon PDGFRα-Cre expression. ECs stained for endomucin are shown in white. Scale = 100 µm. **C,** Confocal co-localization analysis of endogenous fluorescence and immunofluorescence for endomucin (EMCN) that labels endothelial cells in wholemounts shows no co-localization of mesenchymal cells (GFP) with either tdTomato or EMCN (10 FOV). **D,** Immunohistochemistry for YAP shows deletion in head mesenchyme at E12.5 (black square area shown in magnification). Note that ECs still stain positive for YAP in cKO (black arrow). In the cKO the asterisk marks an area of hemorrhage. Scale = 100 µm. **E,** Immunofluorescence for pericyte markers Desmin and NG-2, combined respectively with GFP (antibody) which labels PDGFRa+ cells and endomucin (EMCN) on FFPE tissue from PDGFRa-Cre+ R26mTmG embryos at E11.5. Scale = 100 µm. **F,** Transmission electron microscopy images of wild-type and YAP1 cKO embryos. EC indicates endothelial cell, FIB indicates fibroblast/mesenchymal cell. Coloured boxes indicate the regions selected for higher magnification presentation. Scale = 5 µm, 2 µm, and 500 nm in low, mid, and high magnification panels, respectively. **G,** Pie charts show the relative level of interaction between ECs and fibroblasts. 26 control and 30 cKO images were scored from 3 control and 3 cKO mice. * indicates $p<0.05$ Chi-squared test. **H,** Quantification of branching angle of head vasculature from confocal wholemount data of E10.5 embryos stained for endomucin using machine learning approaches. **I,** Quantification of proportion of vessels in head vasculature with diameters above 60µm for CNT (n=4, purple) and cKO (n=3, blue) from confocal wholemount data of E10.5 embryos stained for endomucin using machine learning approaches (Welch's t test, ** $P < 0.01$).

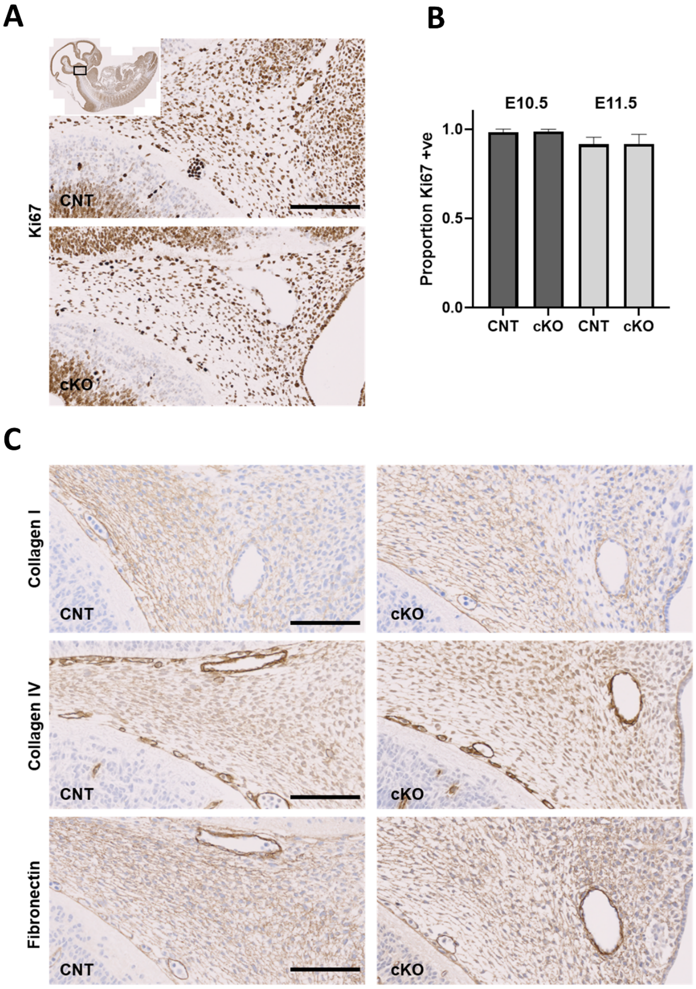

Figure S10: Loss of YAP1 in fibroblasts does not affect proliferation and ECM markers. A, Immunohistochemistry for proliferation marker Ki67 in head region of interest of control (CNT) and YAP cKO (cKO) embryos at E10.5. Scale = 100 µm. **B,** Quantification of Ki67 positive (+ve) cells in head region of interest of control (CNT) and YAP cKO (cKO) embryos at E10.5 and E11.5 (N=3 embryos per condition and embryonic stage) shows no significant differences between CNT and cKO embryos. **C,** Immunohistochemistry for Collagen I, Collagen IV and Fibronectin in head region of interest of control (CNT) and YAP cKO (cKO) embryos at E10.5 shows no significant differences between CNT and cKO embryos. Scale = 100 µm.

Supplementary Tables

Table S1

P values for figure 1G

Day 1	MC	PCC	FB
PCC	1		
FB	3.27E-06	0.00114	
JCC	4.46E-08	6.54E-05	1
**Day 4**	MC	PCC	FB
PCC	5.75E-10		
FB	1.32E-09	1	
JCC	7.43E-37	4.72E-06	3.76E-07
**Day 7**	MC	PCC	FB
PCC	0.00068		
FB	2.98E-21	4.26E-07	
JCC	9.95E-51	1.08E-22	0.00121

Table S2

P values for figure 2 F-I

Length	Day 4	Day 7	Day 10
Day 7	0.86899		
Day 10	0.0203	0.84514	
Day 14	0.22243	1	1
**Perfusability**	Day 4	Day 7	Day 10
Day 7	0.00404		
Day 10	6.54E-04	1	
Day 14	1.83E-05	0.30022	1
**Permeability**	Day 4	Day 7	Day 10
Day 7	0.00466		
Day 10	0.00367	1	
Day 14	0.00464	1	1
**Elastic modulus**	Day 4	Day 7	Day 10
Day 7	6.88E-16		
Day 10	5.11E-25	0.03086	
Day 14	1.68E-14	1	0.13244

Table S3

	Genotype PDGFRa-Cre; Yap1$^{fl/+}$ x Yap1$^{fl/fl}$ cross				
Stage	Yap1$^{fl/+}$	Yap1$^{fl/fl}$	PDGFRa-Cre+ Yap1$^{fl/+}$	PDGFRa-Cre+ Yap1$^{fl/fl}$	Total # of mice
**Weaned mice**	30.5 (25)	28.7 (25)	40.8 (25)	0.0 (25)	174
**E12.5**	26.8 (25)	20.5 (25)	24.1 (25)	28.6 (25)	112
**E11.5**	23.2 (25)	26.1 (25)	22.5 (25)	28.2 (25)	142
**E10.5**	19.6 (25)	22.0 (25)	25.8 (25)	32.5 (25)	209
**E9.5**	19.3 (25)	26.3 (25)	24.5 (25)	29.8 (25)	57

Values in percentage; expected Mendelian ratio in brackets

Supplementary Notes

Lumen formation and Fibroblast colocalization

The mechanism of vascular lumen formation at the cellular level was observed using high resolution, 3D confocal imaging of the JCC system (Fig. S2A). As early as day 1, the formation of intracellular vacuoles was observed in ECs. Multiple vacuoles within an individual cell then coalesced until all the cytoplasm was contained within a thin layer surrounding the large intracellular vacuole. By day 4, we observed the formation of intercellular connections among ECs through which the intracellular vacuoles of multiple cells were joined together and enclosed by the growing surface. Finally, by day 7, a well-defined multi-cellular vessel structure was observed, consisting of a thin EC wall surrounding a clearly defined and fully connected lumen interior. During this time, we also observed FBs elongating along the outer surface of the microvessels (Fig S3A-C) and we measured an increase in colocalization of the two cell types (Fig S3D-E).

Impacts of conditioned media

We sought to determine whether the formation of functional vasculature in the JCC condition might be due primarily to the production and secretion of bioactive molecules resulting from direct interactions between ECs and fibroblasts. To this end, we performed conditioned medium experiments employing the 3-channel fluidic device (Fig. S4). Conditioned medium (CM) was prepared by encapsulating fibroblasts alone (cm FB) or co-culture ECs and fibroblasts (cm JCC) within a fibrin gel in the vascularization channel and harvesting the CM from the medium channels daily. The harvested medium was mixed with fresh cell culture medium at a 1:1 ratio to ensure replenishment of depleted nutrients. Separate experimental devices were prepared with either ECs alone (MC) or co-culture ECs and fibroblasts (JCC) encapsulated in the vascularization channel. Freshly made CM was delivered to the medium channels of these experimental devices daily. Confocal images taken on day 7 of culture showed that only poorly connected, sheet-like structures formed in the MC devices fed with CM (Fig. S4A). We then performed perfusion experiments with fluorescent tracer dye to assess their functionality. Unlike control vasculatures (JCC devices fed with either growth medium or CM), none of the sheet-like structures formed in the CM-fed devices were perfusable, i.e. dextran filled the extracellular fibrin gel in the vascularization channel but not the sheet-like structures. Notably, the formation of functional vasculature in CM-fed JCC devices demonstrated that the lack of formation of functional vasculature in the MC devices fed with CM was not due to the shortage of nutrients. While network length progressively and dramatically increased in JCC systems during vascular morphogenesis, MC devices fed with CM showed no meaningful increase in the network length with time (Fig. S4B). Taken together, these results support the hypothesis that soluble factors, secreted by fibroblasts, alone are not sufficient to achieve optimal functionality, which only occurred when fibroblasts were encapsulated together with ECs in the JCC condition.

Impact of cell density

Although MC constructs supplied with various conditioned media lacked connectivity (Fig. S4A), we found that the paracrine co-culture (PCC) condition - in which ECs and fibroblasts are spatially separated by a medium channel but able to communicate through secreted soluble factors - resulted in a significant increase in network length compared to MC (Fig. 1C). This implies that soluble factors are important for vascular morphogenesis, but potentially require dynamic reciprocal communication between the two cell types. Even so, soluble factor communication alone proved insufficient to achieve optimal network connectivity as evidenced by the fact that JCC conditions still produced superior vascular networks (Fig. 1B-E).

To examine the possibility that varying the numbers of either ECs or fibroblasts in the PCC system might improve vascular morphogenesis to the level found within the JCC system, for example via the increased secretion of growth factors, we measured the effect of increasing the number of ECs or fibroblasts in the devices. We found no significant enhancement in the vascular network length by changing the ratio of ECs to fibroblasts in PCC systems (Fig. S4C, D).

The final concentrations of HUVECs and NHLFs in the JCC system were chosen as 4 × 10^6 /mL and 2 × 10^6 /mL, respectively. Additional experiments revealed that a lower concentration of fibroblasts (10^6 /mL, JCC X=4, where X is the ratio of ECs to fibroblasts) supported vasculature formation to a lesser extent than a higher density of fibroblasts (2 × 10^6 /mL, JCC X=2, Fig. S4F, G). Conversely, increasing the seeding density of fibroblasts (4 × 10^6 /mL, JCC X=1) resulted in the deterioration of the fibrin hydrogel due to excessive contractile forces exerted by the fibroblasts (Fig. S4E). We maintained a constant ECs seeding density of 4 × 10^6 /mL as this density was minimally sufficient for the formation of fully functional networks in the JCC condition, whereas lower densities did not yield fully connected vasculature. Using this density as a baseline, we compared morphogenesis, functional features, and mechanical factors across different conditions and perturbations.

Computational modelling of force-mediated tissue stiffening

To better understand the mechanisms involved in tissue stiffening due to active cell-generated forces, we developed a computational model using a constitutive formulation developed by Shenoy et al. (44). The model is applicable to describe the mechanical behavior of materials with passive and active properties (Fig. S5C). The passive element describes deformation of the tissue under external forces while the active element enables intrinsic generation of contractile forces interacting within the tissue and its surrounding environment (Fig. S5C). To consider the passive behavior, the model uses two parameters (bulk modulus, K, and shear modulus, μ) which were determined from the Young moduli of the decellularized tissue in our system and assuming a Poisson ratio of 0.3 (70). To accommodate the active element, the model requires three more parameters (motor density in the quiescent state, ρ_0, chemical stiffness, β, and chemo-mechanical feedback parameter, α). We adjusted the ρ_0 by comparing the levels of strains released after the cytochalasin D treatment (Fig. 3H). β and α represent the ability of tissues to generate contractile forces and are related to mechanisms that regulate the engagement of motors and stress-dependent signaling pathways, respectively. We obtained these two parameters by running simulations and matching the parameters to achieve the experimentally measured stiffness of the tissue in JCC and Cytochalasin D treated conditions (Fig. 3A). Simulations were run using a commercial finite element software (ABAQUS) and implementing the constitutive model as a UMAT. Using this computational framework, the mechanical response of the tissue was investigated in response to the indentation tests and force-displacement curves were extracted from the model for different values of α (Fig. S5E). Finally, the apparent elastic modulus of the tissue was calculated for each curve using Hertz contact fitting (Fig. S5F). Fig. S5F shows that an increase in the α (or tissue ability to generate active force) leads to an increase in the apparent Young's modulus.

Formulation of the model for 3D condition has been provided previously by Shenoy et al. (44), briefly:

$$\sigma_{ij} = \frac{1}{3}\sigma_{kk}\delta_{ij} + \tilde{\sigma}_{ij} \qquad \rho_{ij} = \frac{1}{3}\rho_{kk}\delta_{ij} + \tilde{\rho}_{ij}$$
$$\sigma_{kk} = 3\rho_0 + 3\bar{K}\varepsilon_{kk} \qquad \rho_{kk} = 3\bar{\rho}_0 + 3\bar{K}_\rho\varepsilon_{kk} \qquad \text{(s1)}$$
$$\tilde{\sigma}_{ij} = 2\bar{\mu}\tilde{\varepsilon}_{ij} \qquad \tilde{\sigma}_{ij} = 2\bar{\mu}_\rho\tilde{\varepsilon}_{ij}$$

in which, σ_{ij} is the stress tensor, δ_{ij} is the Kronecker delta and ρ_{ij} represents the contractility tensor. $\sigma_{kk} = \sigma_{11} + \sigma_{22} + \sigma_{33}$ and $\varepsilon_{kk} = \varepsilon_{11} + \varepsilon_{22} + \varepsilon_{33}$ where ε_{ij} is the strain tensor.

$$\bar{\rho}_0 = \frac{\beta\rho_0}{\beta - \alpha} \qquad \bar{K} = \frac{K\beta - {}^1/_3}{\beta - \alpha} \qquad \text{(S2)}$$
$$\bar{\mu} = \frac{\mu\beta - {}^1/_2}{\beta - \alpha} \qquad \bar{K}_\rho = \frac{K\alpha - {}^1/_3}{\beta - \alpha} \qquad \bar{\mu}_\rho = \frac{\mu\alpha - {}^1/_2}{\beta - \alpha}$$

For the axisymmetric condition, the formulation can be simplified as below:

$$\begin{Bmatrix}\sigma_{11}\\ \sigma_{22}\\ \sigma_{33}\\ \sigma_{12}\end{Bmatrix} = \bar{\rho}_0 \begin{Bmatrix}1\\1\\1\\0\end{Bmatrix} + \begin{bmatrix} \bar{K}+\frac{4}{3}\bar{\mu} & \bar{K}-\frac{2}{3}\bar{\mu} & \bar{K}-\frac{2}{3}\bar{\mu} & 0 \\ \bar{K}-\frac{2}{3}\bar{\mu} & \bar{K}+\frac{4}{3}\bar{\mu} & \bar{K}-\frac{2}{3}\bar{\mu} & 0 \\ \bar{K}-\frac{2}{3}\bar{\mu} & \bar{K}-\frac{2}{3}\bar{\mu} & \bar{K}+\frac{4}{3}\bar{\mu} & 0 \\ 0 & 0 & 0 & \bar{\mu} \end{bmatrix} \begin{Bmatrix}\varepsilon_{11}\\ \varepsilon_{22}\\ \varepsilon_{33}\\ \gamma_{12}\end{Bmatrix} \qquad \begin{Bmatrix}\rho_{11}\\ \rho_{22}\\ \rho_{33}\\ \rho_{12}\end{Bmatrix} = \bar{\rho}_0 \begin{Bmatrix}1\\1\\1\\0\end{Bmatrix} + \begin{bmatrix} \bar{K}_\rho+\frac{4}{3}\bar{\mu}_\rho & \bar{K}_\rho-\frac{2}{3}\bar{\mu}_\rho & \bar{K}_\rho-\frac{2}{3}\bar{\mu}_\rho & 0 \\ \bar{K}_\rho-\frac{2}{3}\bar{\mu}_\rho & \bar{K}_\rho+\frac{4}{3}\bar{\mu}_\rho & \bar{K}_\rho-\frac{2}{3}\bar{\mu}_\rho & 0 \\ \bar{K}_\rho-\frac{2}{3}\bar{\mu}_\rho & \bar{K}_\rho-\frac{2}{3}\bar{\mu}_\rho & \bar{K}_\rho+\frac{4}{3}\bar{\mu}_\rho & 0 \\ 0 & 0 & 0 & \bar{\mu}_\rho \end{bmatrix} \begin{Bmatrix}\varepsilon_{11}\\ \varepsilon_{22}\\ \varepsilon_{33}\\ \gamma_{12}\end{Bmatrix} \tag{S3}$$

where $\gamma_{12} = 2\varepsilon_{12}$.

Perturbation of mechanotransduction pathways in both ECs and fibroblasts

To test whether and how mechanotransduction programs mediate morphogenesis and functional behavior of the vasculature, we exposed JCC constructs to well-established chemical inhibitors (acting on both ECs and fibroblasts) of YAP. Prior to experiments, we performed cell viability and proliferation assays over 4 days of treatment to optimize the concentration of the inhibitors such that, while being effective (and in line with concentrations reported in the literature) the changes in cell viability and proliferation were minimal compared to untreated or DMSO-treated cells (Fig. S7A, B). When cells were treated with 0.25 µM of verteporfin, a YAP-inhibitor, the viability and proliferation were consistently similar to untreated cells (Fig. S7A, B). Therefore, these concentrations were used for all further experiments.

Treatment of JCC constructs with the inhibitor showed that initially rounded and mono-dispersed ECs began to elongate on day 1 (Fig. S7C). However, the inhibition of YAP fully arrested ECs ability to form connected vasculature, in contrast to control conditions which formed tightly interconnected networks by day 4. To investigate the functional impact of inhibitors, we performed perfusion studies on day 7 which revealed that, unlike controls, the vasculature formed in YAP-inhibitor-treated constructs was not perfusable (Fig. S7C).

Transcriptional changes associated with vasculogenesis in fibroblasts and ECs.

To investigate reciprocal interactions between ECs and fibroblasts we performed transcriptomic analysis at various stages of vascular morphogenesis and compared them to mono-culture conditions. Using Principle Component Analysis (PCA), we observed a sharp phenotypic transition between day 7 to day 10 for ECs (Fig. S6A); in fibroblasts the major transition occurred between days 4 and 7 (Fig. S6B), confirming a transient role of fibroblasts, particularly at the early stages. Interestingly, mono-culture ECs/FBs occupy the extreme positions, suggesting that their phenotypic state associated with initial 3D hydrogel encapsulation is unstable, and that stabilizing interactions are necessary for both cell types for vascular morphogenesis to progress.

Gene Set Enrichment Analysis (GSEA) of transcriptomes of ECs at day 1 vs day 14 in co-culture revealed increased expression of endothelial differential and proliferation genes (Fig. S6C). The lack of viability of ECs after 14 days in mono-culture additionally confirms that signals emanating from fibroblasts promote EC survival.

Gene Set Enrichment Analysis (GSEA) of transcriptomes of fibroblasts in mono- vs co-culture at day 14 revealed increased expression of myofibroblast-associated genes (Fig. S6D). These are indicative of a contractile cell state and are likely to contribute to the cytoskeleton-dependent tissue stiffening. Furthermore, there were significant increases in the expression of genes associated with the mechano-transduction signaling pathways YAP/TAZ, and their upstream SRC-family kinases.

Imagej Macro Scripts

2D – Network Length – Confocal Microscope Images

```
//Input is a a .tif confocal image stack with 2 channels: (1) HUVEC, (2) Dextran;
//Note: only the HUVEC channel is needed for this algorithm.
//User is prompted to open folder containing multiple .tif image stacks for processing
dir = getDirectory("Choose a Directory ");
list = getFileList(dir);
networkLength = newArray();
//For each image stack:
for (j=0; j<list.length; j++) {
	path = dir+list[j];
	open(path);
	run("Split Channels");
//Process HUVEC channel, slice by slice
	selectWindow("C1-" + list[j]);
	rename("HUVEC");
//Standard thresholding algorithm:
		for (i=0; i<nSlices; i++) {
		run("8-bit");
		run("Subtract Background...", "rolling=1000 sliding slice");
		run("Enhance Contrast", "saturated=5");
		for(k=0; k<10; k++) run("Despeckle", "slice");
		run("Gaussian Blur...", "sigma=3.0 slice");
		setAutoThreshold("Triangle");
		setAutoThreshold("Triangle dark");
		getThreshold(lower, upper );
		setThreshold(lower, upper );
		run("Convert to Mask", "only");
		run("Next Slice [>]");
		}
//2D projection
	run("Z Project...", "projection=[Max Intensity]");
//Skeletonize
	run("Skeletonize (2D/3D)");
```

```
//Analyze skeleton
        run("Analyze Skeleton (2D/3D)", "prune=none prune");
        run("Close All");
        a = newArray();
//Calculate longest connected network
        for (i=0; i<nResults; i++) {
                b = getResult("# Branches",i)*getResult("Average Branch Length",i);
                a = Array.concat(a,b);
        }
        Array.getStatistics(a, min, max, mean, std);
        networkLength = Array.concat(networkLength,max);
   close("Results");
   print(networkLength[j]);
}
//Network length is the longest connected network for each image; if no units were
//stored in the original image properties, values will be reported in pixels.
```

2D Perfusability

```
//Input is a .tif confocal image stack with 2 channels: (1) HUVEC, (2) Dextran
//User is prompted to open folder containing multiple .tif image stacks for processing
dir = getDirectory("Choose a Directory ");
list = getFileList(dir);
//For each image stack:
for (j=0; j<list.length; j++) {
if (endsWith(list[j],"tif")) {
        path = dir+list[j];
        open(path);
        run("Split Channels");
        selectWindow("C1-" + list[j]);
        rename("HUVEC");
        selectWindow("C2-" + list[j]);
        rename("Dextran");
//Process HUVEC channel
        selectWindow("HUVEC");
//2D Projection
```

```
    run("Z Project...", "projection=[Max Intensity]");
    run("8-bit");
    run("Subtract Background...", "rolling=1000 sliding slice");
    run("Enhance Contrast", "saturated=10");
    for(k=0; k<5; k++) run("Despeckle", "slice");
    run("Gaussian Blur...", "sigma=3.0 slice");
//Binarize
    setAutoThreshold("Triangle");
    setAutoThreshold("Triangle dark");
    getThreshold(lower, upper );
    setThreshold(lower, upper );
    run("Convert to Mask", "only");

    rename("vessels.tif");
//Define Interstitial Region
    run("Duplicate...", "duplicate");
    rename("interstitial.tif");
    run("Invert");
    run("Create Selection");
    roiManager("add");
//Define Vessel Region
    selectWindow("vessels.tif");
    run("Create Selection");
    roiManager("add");
//Process Dextran Channel
    selectWindow("Dextran");
//2D Projection
    run("Z Project...", "projection=[Average Intensity]");
//Measure average dextran intensity in interstitial region, and use to threshold image
    roiManager("measure");
    int_ave = getResult("Mean", 0);
    setAutoThreshold("Default dark");
    setThreshold(int_ave, 4095);
    setOption("BlackBackground", false);
    run("Convert to Mask");
```

```
//Measure average intensity of binarized dextran image within Vessel Region
//Note: for fully perfused vasculature, average binary intensity in vascular region
//would be 255; for fully non-perfused vasculature, average binary intensity in
//vascular region would be 0

        roiManager("measure");
        Perfusability = getResult("Mean", 3)/255;
        run("Close All");
        roiManager("delete");
        selectWindow("Results");
        run("Close");
        print(Perfusability);
        }
}
//Perfusability is reported as a number between 0 and 1
```

3D Perfusability

```
Perfusability_Array = newArray();
//Input is a .tif confocal image stack with 2 channels: (1) HUVEC, (2) Dextran
//User is prompted to open folder containing multiple .tif image stacks for processing
dir = getDirectory("Choose a Directory ");
list = getFileList(dir);
for (j=0; j<list.length; j++) {
        if (endsWith(list[j],"tif")) {
                path = dir+list[j];
                open(path);
                run("Split Channels");
                selectWindow("C1-" + list[j]);
                rename("HUVEC");

                selectWindow("C2-" + list[j]);
                rename("Dextran");
//Process dextran channel, slice by slice
                for (i=0; i<nSlices; i++) {
                run("8-bit");
```

```
		run("Subtract Background...", "rolling=1000 sliding slice");
		run("Enhance Contrast", "saturated=5");
		for(k=0; k<10; k++) run("Despeckle", "slice");
		run("Gaussian Blur...", "sigma=3.0 slice");
//Threshold and Binarize
		setAutoThreshold("Triangle");
		setAutoThreshold("Triangle dark");
		getThreshold(lower, upper );
		setThreshold(lower, upper );
		run("Convert to Mask", "only");
		run("Next Slice [>]");
		}
//Reslice in XZ-plane
		run("Reslice [/]...", "output=1.000 start=Bottom avoid");
		rename("Dextran_Reslice");
//Process HUVEC channel, slice by slice
		selectWindow("HUVEC");
		for (i=0; i<nSlices; i++) {
		run("8-bit");
		run("Subtract Background...", "rolling=1000 sliding slice");
		run("Enhance Contrast", "saturated=5");
		for(k=0; k<10; k++) run("Despeckle", "slice");
		run("Gaussian Blur...", "sigma=3.0 slice");
//Threshold and binarize
		setAutoThreshold("Triangle");
		setAutoThreshold("Triangle dark");
		getThreshold(lower, upper );
		setThreshold(lower, upper );
		run("Convert to Mask", "only");
		run("Next Slice [>]");
		}
//Reslice in XZ-plane
		run("Reslice [/]...", "output=1.000 start=Bottom avoid");
		run("Invert", "stack");
		rename("HUVEC_Reslice");
```

```
run("Set Measurements...", "area mean");
setSlice(1);
//Identify vessel outlines from HUVEC channel
run("Analyze Particles...", "  show=Outlines include add stack");
//Measure binarized dextran pixel intensity within vessel outlines
selectWindow("Dextran_Reslice");
roiManager("measure");
Total_Area = 0;
Zero_Area = 0;
for (I=0; I<nResults; I++) {
b = getResult("Area", I);
Total_Area = Total_Area + b;
a = getResult("Mean", I);
//If no dextran from thresholded image is found within vessel region, that region is
//considered non-perfusable
if (a == 0) {
Zero_Area = Zero_Area + b;
}
}
Perfusability = (Total_Area - Zero_Area) / Total_Area;
Perfusability_Array = Array.concat(Perfusability_Array, Perfusability);
run("Close All");
selectWindow("Results");
run("Close");
roiManager("delete");
}
}
Array.show("Perfusability",Perfusability_Array)
//Perfusability is reported as a fraction of perfused volume divided by total vascular //volume
```

Barrier Function

```
//Input is a .tif confocal image stack with 2 channels: (1) HUVEC, (2) Dextran
//User is prompted to open folder containing multiple .tif image stacks for processing
dir = getDirectory("Choose a Directory ");
list = getFileList(dir);
```

```
//For each image stack:
for (j=0; j<list.length; j++) {

        if (endsWith(list[j],"tif")) {
                path = dir+list[j];
                open(path);
                run("Split Channels");
                selectWindow("C1-" + list[j]);
                rename("HUVEC");
                selectWindow("C2-" + list[j]);
                rename("Dextran");
//Process HUVEC channel
        selectWindow("HUVEC");
//2D projection
        run("Z Project...", "projection=[Max Intensity]");
        run("8-bit");
        run("Subtract Background...", "rolling=1000 sliding slice");
        run("Enhance Contrast", "saturated=10");
        for(k=0; k<5; k++) run("Despeckle", "slice");
        run("Gaussian Blur...", "sigma=3.0 slice");
//Threshold and binarize
        setAutoThreshold("Triangle");
        setAutoThreshold("Triangle dark");
        getThreshold(lower, upper );
        setThreshold(lower, upper );
        run("Convert to Mask", "only");
        rename("vessels.tif");
        run("Duplicate...", "duplicate");
//Define interstitial region
        rename("interstitial.tif");
        run("Invert");
        run("Create Selection");
        roiManager("add");
//Process dextran channel
        selectWindow("Dextran");
```

```
//2D projection
        run("Z Project...", "projection=[Average Intensity]");
//Measure average intensity of dextran in interstitial region
        roiManager("measure");
        run("Close All");
        roiManager("delete");
        selectWindow("Results");
        }
}
```

3D Binary Reconstruction

```
//Input is a confocal image stack of vasculature filled with fluorescently labeled tracer
//Only the tracer (dextran) channel should be used for this thresholding algorithm
//Processing is performed on each slice individually
for (i=0; i<nSlices; i++) {
        run("8-bit");
        run("Subtract Background...", "rolling=1000 sliding slice");
        run("Enhance Contrast", "saturated=5");
        for(k=0; k<10; k++) run("Despeckle", "slice");
        run("Gaussian Blur...", "sigma=3.0 slice");
        setAutoThreshold("Triangle");
        setAutoThreshold("Triangle dark");
        getThreshold(lower, upper );
        setThreshold(lower, upper );
        run("Convert to Mask", "only");
        run("Next Slice [>]");
}
```

Vascular Density and Vascular Volume Fraction

```
//Input is a .tif confocal image stack of vasculature filled with fluorescently labeled
 //tracer.  Only the tracer (dextran) channel should be used for this thresholding
 //algorithm.
//User is prompted to open folder containing multiple .tif image stacks for processing.
dir = getDirectory("Choose a Directory ");
```

```
list = getFileList(dir);
VolFraction = newArray();
NumLumens = newArray();
for (j=0; j<list.length; j++) {
	if (endsWith(list[j],"tif")) {
	path = dir+list[j];
	open(path);
//For each image stack:
//Process, threshold, and binarize – slice by slice
for (i=0; i<nSlices; i++) {
	run("8-bit");
	run("Subtract Background...", "rolling=1000 sliding slice");
	run("Enhance Contrast", "saturated=10");
	for(k=0; k<5; k++) run("Despeckle", "slice");
	run("Gaussian Blur...", "sigma=3.0 slice");
	setAutoThreshold("Triangle");
	setAutoThreshold("Triangle dark");
	getThreshold(lower, upper );
	setThreshold(lower, upper );
	run("Convert to Mask", "only");
	run("Next Slice [>]");
}
//Identify vascular lumens for each slice in XZ-plane
//Calculate # lumens and % vascularized area for each slice
run("Reslice [/]...", "output=1.000 start=Bottom avoid");
rename("lumens.tif");
run("Invert", "stack");
run("Set Measurements...", "area");
run("Analyze Particles...", "size=50-Infinity display summarize stack");
selectWindow("Results");
run("Close");
selectWindow("Summary of lumens.tif");
IJ.renameResults("Results");
	a = newArray();
	c = newArray();
```

```
//Retrieve % vascularized area for each XZ-slice
//Retrieve # lumens for each XZ-slice
	for (i=0; i<nResults; i++) {
		b = getResult("%Area",i);
		a = Array.concat(a,b);
		d = getResult("Count",i);
		c = Array.concat(c,d);
	}
//Calculate average % vascularized area over all slices
	Array.getStatistics(a, min, max, mean, std);
	VolFraction = Array.concat(VolFraction,mean);
//Calculate average # lumens over all slices and normalize by cross-sectional area
	getDimensions(width, height, channels, slices, frames);
	getPixelSize(unit, pixelWidth, pixelHeight);
	totalarea = (width*pixelWidth*height*pixelHeight)/1000000;
	Array.getStatistics(c, minc, maxc, meanc, stdc);
	NumLumens = Array.concat(NumLumens,meanc/totalarea);
	selectWindow("Results");
	saveAs("Text", path + "Morph_Quant.txt");
	run("Close All");
	}
}
Array.show("VolFraction",VolFraction)
Array.show("NumVessels",NumLumens)
```

Permeability

```
//Input is a .tif confocal image stack of vasculature filled with fluorescently labeled
//tracer.
//Only the tracer (dextran) channel should be used for this thresholding algorithm.

//***User MUST manually enter the time intervals – in minutes – between the two
//images taken as an array (one for each ROI being processed) – "dt" ***
dt = newArray(12, 12, 12);
//Drift correction:  External drift correction should be applied so that individual stack
 //slices of an ROI match-up at both time points.  If no appreciative drift occurred,
```

```
//enter the full stack range below

//Drift corrected stack sizes (z1 – z2) – t1
z1list = newArray(1, 2, 1);
z2list = newArray(50, 50, 50);
//Drift corrected stack sizes (z1 – z2) – t2
z1list_t2 = newArray(1, 1, 1);
z2list_t2 = newArray(50, 49, 50);

//User is prompted to open folder containing multiple .tif image stacks for processing
dir = getDirectory("Choose a Directory for t1 ");
list = getFileList(dir);
dir_1 = getDirectory("Choose a Directory for t2 ");
list_1 = getFileList(dir_1);
s = 0;
for (j=0; j<list.length; j++) {
	if (endsWith(list[j],"tif")) {
	path = dir+list[j];
	z1 = z1list[s];
	z2 = z2list[s];
	open(path);
	c = " range="+z1+"-"+z2;
	run("Duplicate...", "duplicate"+c);
	rename("original.tif");
	run("Duplicate...", "duplicate");

//For each image stack:
//Process, threshold, and binarize – slice by slice
for (i=0; i<nSlices; i++) {
	run("8-bit");
	run("Subtract Background...", "rolling=1000 sliding slice");
	run("Enhance Contrast", "saturated=5");
	for(k=0; k<10; k++) run("Despeckle", "slice");
	run("Gaussian Blur...", "sigma=3.0 slice");
	setAutoThreshold("Triangle");
```

```
        setAutoThreshold("Triangle dark");
        getThreshold(lower, upper );
        setThreshold(lower, upper );
        run("Convert to Mask", "only");
        run("Next Slice [>]");
}
run("Reslice [/]...", "output=1.000 start=Bottom avoid");
rename("interstitial.tif");
run("Duplicate...", "duplicate");
rename("lumens.tif");
run("Invert", "stack");

//Measure morphological properties
run("Set Measurements...", "area perimeter");
run("Analyze Particles...", "size=1-Infinity display summarize stack");
selectWindow("Summary of lumens.tif");
saveAs("Text", path + "Summary of lumens.txt");
IJ.renameResults("Results");
        aa = newArray();
        bb = newArray();
        for (i=0; i<nResults; i++) {
                b = getResult("%Area",i);
                d = getResult("Count",i);
                f = getResult("Perim.",i);
                h = getResult("Total Area",i);
                aa = Array.concat(aa,((h/(b/100))-h));
                bb = Array.concat(bb,(d*f));
        }

//Calculate Interstitial Volume / Vascular Surface Area
        Array.getStatistics(aa, minaa, maxaa, meanaa, stdaa);
        Array.getStatistics(bb, minbb, maxbb, meanbb, stdbb);
        V_S = meanaa/meanbb;
        print("V / S = " + V_S);
        selectWindow("Results");
```

```
run("Close");

//Define Vascular Region

run("Set Measurements...", "area mean");
selectWindow("lumens.tif");
setSlice(1);
for (l=0; l<nSlices; l++) {
run("Create Selection");
roiManager("add");
run("Next Slice [>]");
}
roiManager("save", path + "lumen_ROI.zip");

//Calculate initial average dextran intensity in vascular region
selectWindow("original.tif");
run("Reslice [/]...", "output=1.000 start=Bottom avoid");
rename("originalReslice.tif");
roiManager("measure");
roiManager("delete");
nn = nResults;
run("Summarize");
Perm_Lumens = getResult("Mean", nn);
print("Perm_Lumens = " + Perm_Lumens);
IJ.renameResults("Lumens");
selectWindow("Lumens");
saveAs("Text", path + "Permeability_Lumens.txt");
run("Close");

//Define interstitial region
selectWindow("interstitial.tif");
setSlice(1);
for (m=0; m<nSlices; m++) {
run("Create Selection");
roiManager("add");
```

```
run("Next Slice [>]");
}
roiManager("save", path + "interstitial_ROI.zip");

//Calculate initial average dextran intensity in interstitial region
selectWindow("originalReslice.tif");
roiManager("measure");
nn = nResults;
run("Summarize");
Perm_Interstitial = getResult("Mean", nn);
print("Perm_Interstitial = " + Perm_Interstitial);
IJ.renameResults("Interstitial");
selectWindow("Interstitial");
saveAs("Text", path + "Permeability_Interstitial.txt");
run("Close");

//Process t2 time-point
path_1 = dir_1+list_1[s];
z1 = z1list_t2[s];
z2 = z2list_t2[s];
open(path_1);
c = " range="+z1+"-"+z2;
run("Duplicate...", "duplicate"+c);
run("Reslice [/]...", "output=1.000 start=Bottom avoid");

////Calculate final average dextran intensity in interstitial region
roiManager("measure");
roiManager("delete");
nn = nResults;
run("Summarize");
Perm_Interstitial_t2 = getResult("Mean", nn);
print("Perm_Interstitial_t2 = " + Perm_Interstitial_t2);
IJ.renameResults("Interstitial_Perm_t2");
selectWindow("Interstitial_Perm_t2");
saveAs("Text", path + "Permeability_Interstitial_t2.txt");
```

```
run("Close");
run("Close All");

//Calculate permeability
permeability = (V_S*((Perm_Interstitial_t2 -Perm_Interstitial)/(dt[s]*60))*(1/(Perm_Lumens - Perm_Interstitial)))/10000;
print("Permeability = " + permeability);
s = s + 1;
}
else {
}
run("Close All");
}
//Permeability is reported in cm/s
```
